# Assessing the Impact of Evidence-Based Mental Health Guidance During the COVID-19 Pandemic: Systematic Review and Qualitative Evaluation

**DOI:** 10.2196/52901

**Published:** 2023-12-22

**Authors:** Katharine A Smith, Edoardo G Ostinelli, Roger Ede, Lisa Allard, Michaela Thomson, Kiran Hewitt, Petra Brown, Caroline Zangani, Matthew Jenkins, Verena Hinze, George Ma, Prajnesh Pothulu, Catherine Henshall, Gin S Malhi, Susanna Every-Palmer, Andrea Cipriani

**Affiliations:** 1 Department of Psychiatry University of Oxford Oxford United Kingdom; 2 Oxford Health NHS Foundation Trust Oxford United Kingdom; 3 Oxford Precision Psychiatry Lab NIHR Oxford Health Biomedical Research Centre Oxford United Kingdom; 4 Pharmacy Department Royal Brisbane and Women’s Hospital Brisbane Australia; 5 Mersey Care NHS Foundation Trust Liverpool United Kingdom; 6 Lincolnshire Partnership NHS Foundation Trust Lincoln United Kingdom; 7 Pennine Care NHS Foundation Trust Manchester United Kingdom; 8 Department of Pharmacy and Optometry University of Manchester Manchester United Kingdom; 9 Department of Psychological Medicine University of Otago Wellington New Zealand; 10 Pharmacy Department The Prince Charles Hospital Metro North Health Brisbane Australia; 11 Pharmacy Department Princess Alexandra Hospital Brisbane Australia; 12 Oxford Institute of Applied Health Research Oxford Brookes University Oxford United Kingdom; 13 Nursing and Midwifery Office National Institute for Health and Care Research London United Kingdom; 14 Academic Department of Psychiatry, Faculty of Medicine and Health Kolling Institute, Northern Clinical School The University of Sydney Sydney Australia; 15 CADE Clinic and Mood-T Royal North Shore Hospital, Northern Sydney Local Health District St. Leonards, New South Wales Australia

**Keywords:** evidence synthesis, guidelines, mental health, systematic review, focus group, survey, COVID-19, pandemic, digital health, eHealth, mobile phone

## Abstract

**Background:**

During the COVID-19 pandemic, the Oxford Precision Psychiatry Lab (OxPPL) developed open-access web-based summaries of mental health care guidelines (OxPPL guidance) in key areas such as digital approaches and telepsychiatry, suicide and self-harm, domestic violence and abuse, perinatal care, and vaccine hesitancy and prioritization in the context of mental illness, to inform timely clinical decision-making.

**Objective:**

This study aimed to evaluate the practice of creating evidence-based health guidelines during health emergencies using the OxPPL guidance as an example. An international network of clinical sites and colleagues (in Australia, New Zealand, and the United Kingdom) including clinicians, researchers, and experts by experience aimed to (1) evaluate the clinical impact of the OxPPL guidance, as an example of an evidence-based summary of guidelines; (2) review the literature for other evidence-based summaries of COVID-19 guidelines regarding mental health care; and (3) produce a framework for response to future global health emergencies.

**Methods:**

The impact and clinical utility of the OxPPL guidance were assessed using clinicians’ feedback via an international survey and focus groups. A systematic review (protocol registered on Open Science Framework) identified summaries or syntheses of guidelines for mental health care during and after the COVID-19 pandemic and assessed the accuracy of the methods used in the OxPPL guidance by identifying any resources that the guidance had not included.

**Results:**

Overall, 80.2% (146/182) of the clinicians agreed or strongly agreed that the OxPPL guidance answered important clinical questions, 73.1% (133/182) stated that the guidance was relevant to their service, 59.3% (108/182) said that the guidelines had or would have a positive impact on their clinical practice, 42.9% (78/182) that they had shared or would share the guidance, and 80.2% (146/182) stated that the methodology could be used during future health crises. The focus groups found that the combination of evidence-based knowledge, clinical viewpoint, and visibility was crucial for clinical implementation. The systematic review identified 2543 records, of which 2 syntheses of guidelines met all the inclusion criteria, but only 1 (the OxPPL guidance) used evidence-based methodology. The review showed that the OxPPL guidance had included the majority of eligible guidelines, but 6 were identified that had not been included.

**Conclusions:**

The study identified an unmet need for web-based, evidence-based mental health care guidance during the COVID-19 pandemic. The OxPPL guidance was evaluated by clinicians as having a real-world clinical impact. Robust evidence-based methodology and expertise in mental health are necessary, but easy accessibility is also needed, and digital technology can materially help. Further health emergencies are inevitable and now is the ideal time to prepare, including addressing the training needs of clinicians, patients, and carers, especially in areas such as telepsychiatry and digital mental health. For future planning, guidance should be widely disseminated on an international platform, with allocated resources to support adaptive updates.

## Introduction

### Background

Globally, the needs of those with mental illness were particularly acute during the COVID-19 pandemic, especially during lockdown periods. COVID-19 highlighted preexisting disparities in health care and the increased risks for those with mental disorders [1,2]. Guidelines specifically for mental health disorders were often limited or difficult to find, particularly in the early phases of the pandemic [3]. As it was primarily a respiratory infection, early COVID-19 guidance often focused on the physical management of patients, whereas guidance for those with mental disorders was largely absent or hidden within the wider recommendations [3]. However, the needs of people with serious mental illness and their difficulties in complying with isolation and distancing regulations (often because of the structural design and layout of psychiatric facilities), in addition to the associated physical, social, and economic disadvantages associated with long-term serious mental health conditions, made this group especially vulnerable to the adverse effects of the pandemic [4].

At the same time, the COVID-19 pandemic also created an opportunity to examine the practice of developing evidence-based health guidelines during a health emergency [5,6]. Substantial progress was made in the rapid generation of new evidence, particularly in physical health care (eg, the RECOVERY and COVID-19 vaccine trials [7]), but there were also examples of multiple research studies and systematic reviews that overlapped or were too small to produce significant findings [8]. Ideally, guidelines, including those for mental disorders, should be an easily accessible resource for clinicians and patients to enable them to improve clinical outcomes [9]. Before the pandemic, there were already established methods for developing guidelines (eg, the GRADE Evidence to Decision frameworks [10]), but in practice, these were often not followed [11]. There are also well-defined methods for rapid review and guideline development, applicable in the context of health emergencies [12-14]. However, the large number of clinical guidelines and consensus statements produced in the early phase of the COVID-19 pandemic were often country or service specific, were focused on physical care, were of variable methodology and quality, or were not regularly updated, often leaving clinicians confused about where to turn [12].

During the COVID-19 pandemic, we (the Oxford Precision Psychiatry Lab [OxPPL]; [15]) developed evidence-based summaries of guidelines regarding mental disorders using a pragmatic, evidence-based approach (“OxPPL guidance”) [3]. This study aimed to complete a formal assessment of the real-world clinical impact of these syntheses of guidelines. We recruited an international network of clinical sites and colleagues (in Australia, New Zealand, and the United Kingdom) to provide feedback from countries and regions that had experienced different case rates and lockdown restrictions throughout the COVID-19 pandemic.

### Study Objectives

Our study objectives were as follows:

1. To assess the impact and clinical utility of the OxPPL guidance, by collecting clinicians’ feedback via an international survey and focus groups
2. To conduct a systematic review of the literature to identify and compare the OxPPL guidance with other syntheses of mental health care guidelines in the context of COVID-19 and to assess the accuracy of the OxPPL guidance methodology
3. To produce a framework for evidence syntheses to support guideline development in mental health care for future global health emergencies

## Methods

This study aimed to assess the real-world impact of the resources developed by OxPPL [15]. The methods used to develop the OxPPL guidance are outlined in detail elsewhere [3] and are summarized briefly for context in this paper.

### Development of Guidance Resources

Starting in March 2020, a multidisciplinary team at OxPPL [15] developed evidence-based summaries of guidelines for managing mental health disorders in the context of COVID-19.

The OxPPL guidance team consisted of mental health researchers (physicians, methodologists, nurses, and pharmacists) who systematically searched English-language websites for guidelines about a range of topics in managing mental illness in the context of the COVID-19 pandemic and synthesized these into summaries of guidance using a validated, evidence-based approach [3]. A “bottom up” as well as “top-down” approach [16] was used: the choice of topics was driven by clinical needs, in consultation with clinician colleagues within local UK National Health Service (NHS) mental health services and with international collaborators. The topics included a range of approaches to the care of patients with mental illness during the COVID-19 pandemic including medications, psychological treatment, organization of services, and modes of delivery. Initially we focused on immediate priority areas. The rapid transition to telepsychiatry services [17,18] prompted the need for guidance syntheses regarding digital approaches and telepsychiatry [19], followed by other areas of mental health including inpatient care; use of clozapine, lithium, and antipsychotics; suicide and self-harm; domestic violence and abuse; substance use disorders [20]; perinatal care [21]; and vaccine prioritization [22] and hesitancy [23] in the context of mental illness. The team updated the guidance regularly and collaborated with experts in each area to keep the guidance focused, comprehensive, and globally representative. The OxPPL guidance [24] was free to access and advertised via NHS websites, social media, and academic and clinical contacts. The guidance team also collaborated with other sites to adapt and translate the guidance for use in 6 non–English-speaking countries (China, Italy, Bulgaria, France, Japan, and Turkey).

### Recruitment of Sites to Collaborate in Assessing the Impact of the OxPPL Guidance

We recruited a multidisciplinary international network of clinical sites and colleagues (including clinicians, researchers, and experts by experience), with 4 sites across the United Kingdom, 2 sites in Australia, and 1 site in New Zealand. Collaborators from the sites participated in the systematic review and the development of the survey, including providing adapted versions for clinicians in Australia and New Zealand. They also identified and facilitated the routes for dissemination of the survey to patient-facing clinicians in their mental health care services and identified potential participants for the focus groups.

### Development of the Survey

A multidisciplinary group involving clinicians from medicine, nursing, psychology, and pharmacy and a Patient and Public Involvement representative developed the survey using an iterative process. The focus of the survey was to collect multisite, multidisciplinary, international feedback about the OxPPL guidance in its current format, its usefulness during the acute phase and immediate aftermath of the pandemic, and any potential uses and adaptations for future use.

Different versions of the survey were directed to respondents if they had or had not seen the OxPPL guidance before completing the survey. Those who had not seen the guidance previously were invited to do so before answering the survey questions. The main survey was created by the multidisciplinary group (based in the United Kingdom), and collaborators from the Australia and New Zealand sites created the adapted versions, which were consistent with local practices. Key changes for Australia and New Zealand were to use country-specific ethnicity categories and descriptions of mental health service backgrounds.

The survey was hosted on Microsoft Forms, and a link was sent with a covering email to all the clinicians in mental health care within the participating sites inviting a response, and follow-up reminder emails were sent after this. This process was coordinated by the lead at each site using existing email databases, which included (but were not necessarily limited to) staff members within relevant mental health services. The survey and email specified that completion was restricted to patient-facing mental health care staff. In addition, any respondents who answered *no* to the initial screening question within the survey about this were redirected and thanked for their time, and no further survey completion was allowed. The survey was open for 3 months (from September 13, 2022, to December 25, 2022).

The survey (copy available on request from the authors) was anonymous but collected demographic data (eg, age, gender, ethnicity, place of work, and professional background) for descriptive purposes. All participants were asked which topics they had looked at or used, whether they thought that the guidance answered important clinical questions, whether the methods used were appropriate, whether the layout was easy to access, and whether the extra features (eg, downloadable summaries) were useful. Respondents were also asked about the real-world impact of the OxPPL guidance: whether it had made an impact on their clinical practice; whether it was relevant and applicable in their work setting and patient population; and whether they had shared the OxPPL guidance with other clinicians, patients, or carers. Answers were scored on a 5-point Likert scale ranging from strongly disagree to strongly agree.

### Ethical Considerations

The participating sites obtained ethics and locality approval for the study, as required under local governance. The 4 UK sites gained approvals as service evaluations from their local UK NHS Trusts. Ethics approval in Australia was obtained from the Gold Coast Hospital and Health Service (ethics EX/2022/QGC/87527) and in New Zealand from the University of Otago Ethics Committee (reference 22/103), with Whatu Ora locality approval and Māori Consultation with the Ngāi Tahu Research Committee. Participants in the focus groups received recompense for their time (£15, approximately US $19, or an equivalent voucher) after completion of the group.

### Focus Groups

The aim of the focus groups was to provide more detailed information about clinicians’ views regarding the OxPPL guidance. Further details are available in Multimedia Appendix 1, but the broad topic prompts included a short opening section with prompts to discuss participants’ work settings, experiences during the COVID-19 pandemic, and general routes of information seeking for the management of mental illness. Most of the group’s time focused on the use of the OxPPL guidance. The facilitator followed the topic guide and there were broad prompts encouraging feedback about the OxPPL guidance resources, including clinical relevance, ease of use, reliability, and areas for improvement. Finally, there was a discussion about its applicability to future health crises or pandemics.

In total, 2 focus groups were conducted at different times to allow for international participation across different time zones and work patterns. Participants from a range of professional backgrounds within the participating mental health services were invited, and recruitment was through convenience sampling. Participants received a participant information sheet and completed written informed consent before the group, with the opportunity to ask further questions if needed. The focus groups lasted for an hour and were facilitated by 2 researchers (CH and KAS) using a semistructured topic guide (Multimedia Appendix 1). The groups were conducted remotely, recorded, and transcribed. The data were analyzed thematically and managed using the Framework method [25], with double coding of transcripts by 2 researchers (KAS and CH) to ensure consistency. Once the researchers had coded and categorized the data within the Framework matrix, the team discussed any emerging findings, to aid interpretation and explore and develop themes relating to participants’ views and experiences. The COREQ (Consolidated Criteria for Reporting Qualitative Research) guidelines [26] were used to report the qualitative results.

### Systematic Review

The primary aim of the systematic review was to assess whether there were other reported syntheses of guidance in the management of mental illness during the COVID-19 pandemic. A secondary aim was to assess the accuracy of the methodology used in the OxPPL guidance to include all the available individual resources—this was completed by also extracting all the papers about individual resources, which met the criteria for inclusion in the OxPPL guidance, and comparing this with the group of resources actually included.

The protocol for the systematic review with full details was published on Open Science Framework [27], and we have reported the results following the PRISMA (Preferred Reporting Items for Systematic Reviews and Meta-Analyses) guidelines [28]. The completed PRISMA checklist is available in Multimedia Appendix 2. The search strategy included broad terms relating to mental health, the COVID-19 pandemic, and guidelines or guidance, to capture all the available records (for full details, refer to the protocol in Open Science Framework [27]) from database inception until the search date (March 22, 2023).

At least 2 members of the review team (KAS, SE-P, and GSM) independently screened the title and abstract of the retrieved records. Full texts of the potentially eligible records were then assessed against the eligibility criteria by 2 researchers (KAS and EGO). Any disagreement was discussed with another member of the research team (AC). Overall, three main groups of papers were considered to be eligible: those describing (1) syntheses or (2) collections of guidelines regarding mental illness and COVID-19, and (3) papers reporting individual guidelines that met the criteria for inclusion in the OxPPL guidance were also retrieved, and their content was compared against the OxPPL guidance. We included only reports that were relevant to a diagnosis of mental illness (therefore, we did not include reports solely related to the prevention of mental health symptoms or the well-being of health care staff or the general public). Guidelines related to mental disorders following the acute pandemic (eg, post–COVID-19 condition [long COVID], the neuropsychiatric consequences of COVID-19) were not included. Data extraction from the included reports was performed and double checked by 2 researchers (KAS and EGO), including the countries covered by the guidelines, date, methodology, type (synthesis, collection, or individual guideline), and key findings.

## Results

### Characteristics of the Sites Participating in the Survey and Focus Groups

**Figure 1:**
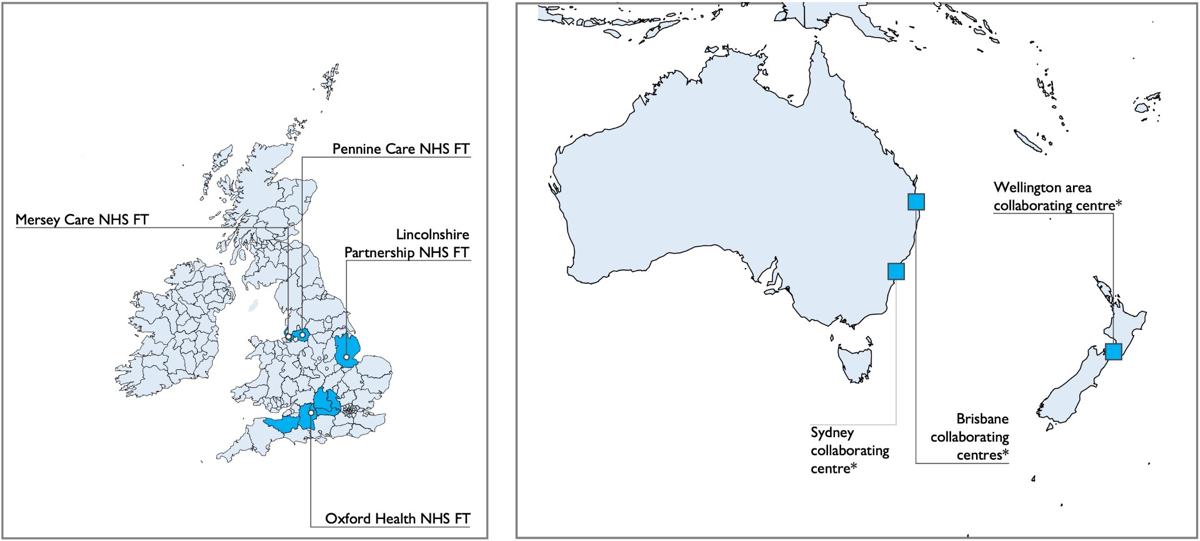
Participating sites and their locations. NHS FT: National Health Service Foundation Trust; *Brisbane collaborating centers: Royal Brisbane and Women’s Hospital; Prince Charles Hospital; and Princess Alexandra Hospital, Brisbane, Queensland (survey only); *Sydney collaborating center: Royal North Shore Hospital, Sydney, New South Wales (focus group only); *Wellington area collaborating center: Mental Health, Addiction and Intellectual Disability Service, Wellington and surrounding areas, North Island. This figure was created using MAPSVG [29] which is licensed under Creative Commons Attribution 4.0 International License [30].

Characteristics and locations of the 6 participating sites are shown in Table 1 and Figure 1. There were 4 sites in the United Kingdom (in England, across different areas of the country); 2 sites in Australia (Sydney [focus groups only] and Brisbane [survey only]); and 1 in Wellington, New Zealand.

**Table 1:** Characteristics of the participating sites.

Site^a^	Staff who were sent the survey link (N=17,473), n (%)	Further information
Mersey Care NHS^b^ Foundation Trust, United Kingdom	9028 (51.67) patient-facing mental health staff	This NHS Trust provides physical health and mental health services in the North West of England, serving >1.4 million people. It is also commissioned for services that cover the North West, North Wales, and the Midlands. Core centers are in Liverpool, Sefton, Knowsley, St Helens, Halton, and Warrington. It provides specialist inpatient and community services to support physical and mental health and specialist inpatient mental health, learning disability, addiction, and brain injury services. It is 1 of 3 NHS Trusts in the United Kingdom that offer high-security mental health facilities.
Pennine Care NHS Foundation Trust, United Kingdom	3083 (17.64) mental health and learning disabilities staff	This is an NHS Trust in the North of England, serving a population of 1.3 million in 6 boroughs: Bury, Glossop, Oldham, Rochdale, Stockport, and Tameside.
Lincolnshire Partnership NHS Foundation Trust, United Kingdom	1770 (10.13) patient-facing mental health staff	This NHS Trust in the East of England provides services to a population of 766,000 in Lincolnshire and 160,000 in North East Lincolnshire. Main sites are Lincoln, Grantham, and Boston. It includes community mental health teams and several other specialist, crisis, and home treatment services and inpatient beds.
Oxford Health NHS Foundation Trust, United Kingdom	2113 (12.09) mental health staff in the Oxfordshire and Buckinghamshire Mental Health directorates	This NHS Trust provides community health, mental health, and specialized health services to approximately 2 million people in an area in the Southeast of England across the counties of Oxfordshire, Buckinghamshire, Berkshire, Wiltshire, Swindon, Bath, and North East Somerset. It also provides a range of specialized health services including forensic mental health and eating disorder services across a wide geographic area including support for patients in Berkshire and Wales.
Brisbane, Australia	830 (4.75) patient-facing mental health staff	Staff approached were from mental health services within Metro South Health and Metro North Health, Brisbane, Queensland, including the Royal Brisbane and Women’s Hospital, Prince Charles Hospital, and Princess Alexandra Hospital. Staff worked within a variety of services including acute adult inpatients, adult community mental health (outpatients), older persons mental health, mobile intensive and long-term rehabilitation, early psychosis mental health, adolescent mental health, homeless outreach team, psychiatric emergency care, and alcohol and drug services.
Wellington, New Zealand	649 (3.71) patient-facing mental health clinicians	Staff were surveyed from the Mental Health, Addiction, and Intellectual Disability Service, which serves the lower North Island of New Zealand and includes local, regional, and national services. Services are provided from multiple sites within greater Wellington, Hutt Valley, and Wairarapa. The service covers a range of specialties in mental health including adult, community, crisis, consult liaison, child and adolescent, older adult, forensic, eating disorders, and substance misuse.

^a^Sydney, Australia, site participated in the focus group but not in the survey.

^b^NHS: National Health Service.

### Survey

Response rates to the survey were low. From a total of 17,473 staff who were emailed across all the sites, only 184 (1.05%) survey responses were received. In country-specific comparisons, response rates were higher in New Zealand (48/649, 7.4%), but the absolute numbers were small.

#### Characteristics of the Survey Participants

Characteristics of the 184 survey participants are shown in Table 2 and Multimedia Appendix 3. The clinical staff who completed the survey had a range of professional backgrounds including nursing (74/184, 40.2%), allied health professions (58/184, 31.5%), and medical (37/184, 20.1%). Within mental health, different services were represented, with 52.2% (96/184) working in adult mental health. Of the 184 participants, 120 (65.2%) were women and 164 (89.1%) were aged between 25 and 64 years. Most participants in the United Kingdom identified their ethnic group as White (British, Irish, or other; 97/120, 80.8%), most of those in New Zealand identified as New Zealand European (25/48, 52%) or White (2/48, 4%), and most of those in Australia identified as Oceanian (7/16, 44%) or White (2/16, 13%). Other ethnic backgrounds were represented but in much smaller numbers. For example, in the United Kingdom, 3.3% (4/120) identified as Black or Black British, and in New Zealand, 15% (7/48) identified as Māori or Māori-New Zealand European.

**Table 2:** Characteristics of the survey participants.

Characteristics		United Kingdom (n=120), n (%)	New Zealand (n=48), n (%)	Australia (n=16), n (%)	Total (n=184), n (%)
**Professional background**					
	Medical	28 (23.3)	8 (17)	1 (6)	37 (20.1)
	Nursing	46 (38.3)	24 (50)	4 (25)	74 (40.2)
	Occupational therapy	1 (0.8)	3 (6)	1 (6)	5 (2.7)
	Pharmacy	6 (5)	0 (0)	3 (19)	9 (4.9)
	Physiotherapy	2 (1.7)	0 (0)	0 (0)	2 (1.1)
	Psychology	14 (11.7)	9 (19)	2 (13)	25 (13.6)
	Social work	10 (8.3)	2 (4)	5 (31)	17 (9.2)
	Other	13 (10.8)	2 (4)	0 (0)	15 (8.2)
**Main service (>50% of the time)**					
	Adult mental health	65 (54.2)	18 (38)	13 (81)	96 (52.2)
	Child and adolescent mental health	9 (7.5)	12 (25)	1 (6)	22 (12.0)
	Forensic mental health	12 (10)	13 (27)	1 (6)	26 (14.1)
	Learning disability	6 (5)	0 (0)	0 (0)	6 (3.3)
	Memory assessment clinic	3 (2.5)	0 (0)	0 (0)	3 (1.6)
	Older adult mental health	7 (5.8)	2 (4)	1 (6)	10 (5.4)
	Not related to mental health	2 (1.7)	0 (0)	0 (0)	2 (1.1)
	Other	16 (13.3)	3 (6)	0 (0)	19 (10.3)
**Age group (years)**					
	18-24	9 (7.5)	0 (0)	0 (0)	9 (4.9)
	25-34	29 (24.2)	14 (29)	3 (19)	46 (25)
	35-44	24 (20)	11 (23)	6 (38)	41 (22.3)
	45-54	34 (28.3)	10 (21)	3 (19)	47 (25.5)
	55-64	16 (13.3)	10 (21)	4 (25)	30 (16.3)
	65-74	5 (4.2)	0 (0)	0 (0)	5 (2.7)
	≥75	0 (0)	2 (4)	0 (0)	2 (1.1)
	Prefer not to say or N/A^a^	3 (2.5)	1 (2)	0 (0)	2 (1.1)
**Gender**					
	Female	78 (65)	31 (65)	11 (69)	120 (65.2)
	Male	39 (32.5)	16 (33)	5 (31)	60 (32.6)
	Nonbinary	1 (0.8)	0 (0)	0 (0)	1 (0.5)
	Prefer not to say or N/A	2 (1.7)	1 (2)	0 (0)	3 (1.6)

^a^N/A: not applicable.

#### Survey Responses

Survey responses are described in Multimedia Appendix 4. Of the 184 who started the survey, 2 (1.3%) responded that they did not work within mental health, and therefore could not complete the subsequent mental health guidance questions. Overall, 56.6% (103/182) reported not having seen the OxPPL guidance before the survey, with higher rates in both Australia and New Zealand than in the United Kingdom. Of those who were already aware of the resources (ie, had seen them), the more frequently accessed topics were suicide and self-harm (accessed by 38/79, 48%) and vaccine uptake and hesitancy (accessed by 34/79, 43%). Overall, 35% (28/79) had looked at telepsychiatry and digital approaches. Of those who looked at this guidance for the first time within the study, more frequently accessed topics were also suicide and self-harm (38/103, 36.9%) and clozapine treatment (35/103, 34.0%). Furthermore, 23.3% (24/103) had looked at telepsychiatry and digital approaches.

Of the survey respondents, 80.2% (146/182) agreed or strongly agreed that the OxPPL guidance answered important clinical questions, 76.4% (139/182) stated that the methods used were appropriate (ie, sufficiently trustworthy to be used in clinical practice), 78% (142/182) said that the web-based layout was easy to access, and 79.7% (145/182) stated that the extra web-based features (eg, downloadable summaries) were useful. Overall, 73.1% (133/182) reported that the guidance was relevant and applicable for their service, and 72% (131/182) that it was relevant and applicable for their patient population. Overall, 59.3% (108/182) reported that the guidelines had or would have a positive impact on their clinical practice, and 42.9% (78/182) reported that they had shared or would share the guidance with others. For those who had already shared the guidance, 90% (70/78) had shared with coworkers, 33% (26/78) with other professionals, 21% (16/78) with patients, and 12% (9/78) with carers.

Furthermore, 73.1% (133/182) of the survey respondents reported having also used other resources for information including specialty-based, profession-based, and governmental websites. However, 26.9% (49/182) reported having used no other resource for guidance in mental health diagnosis and treatment during the COVID-19 pandemic. Overall, 80.2% (146/182) agreed or strongly agreed that the methodology used by the OxPPL guidance could be used in future pandemics and health crises, and 64.3% (117/182) stated that other topics could then be added to meet these future needs.

### Focus Groups

The 2 focus groups included a total of 18 participants (n=8, 44% women), including 9 (50%) physicians, 2 (11%) pharmacists, 3 (17%) nurses, 1 (6%) occupational therapist, 2 (11%) health care assistants, and 1 (6%) social worker. The participants worked across different areas of mental health including adult, child and adolescent, specialist bipolar disorder, forensic, and eating disorders (Multimedia Appendix 5).

The main themes, with illustrative quotes arising from the framework analysis of the content of the focus groups are summarized in Table 3, and the full Framework analysis is presented in Multimedia Appendix 6.

In total, 4 main themes emerged from the analysis. These were (1) challenges and uncertainty during the pandemic, (2) need for trustworthy information, (3) feedback about the OxPPL guidance, and (4) use of the OxPPL guidance in the future. In theme 1, many participants commented about the challenges and uncertainties faced by mental health clinicians and patients during the pandemic. Participants identified the constantly changing nature of the challenges, high workload, staff shortages, changing staff roles, and the pressure and urgency to implement changes. The rapid transition to telepsychiatry was highlighted as a key change which required adaptation. It was also noted that mental health often seemed to be forgotten, especially in the early stages, but that mental health care had particular challenges in the context of the pandemic, including in inpatient care. Participants from New Zealand and Australia reflected about their different experiences compared with those in the United Kingdom and other countries. Theme 2 explored the need for formal guidance in mental health care: participating clinicians commented that they felt their own uncertainty had affected patients. They also noted that patients often felt more uncertain because of their mental health issues, which led to seeking information from unreliable sources. Participants reported uncertainty about decision-making and noted that there had been many complex decisions in mental health settings. They also described their view that there was an unmet need for reliable and trustworthy guidance to support their decision-making. Theme 3 explored a discussion about the OxPPL guidance: most participants had not been aware of this before the group but reported that they wished they had seen it sooner. Areas such as layout, the web-based open-access format, range of topics, ease of sharing with colleagues, and suitability for different professionals received positive feedback. The combination of evidence-based methods and clinical relevance was felt to be essential. Theme 4 focused on future uses: increased visibility was a key area, new topics were suggested, and possible modifications for patient use were discussed. The participants felt that COVID-19 would continue to be an issue and that the OxPPL guidance would continue to be relevant. The resources were felt to be relevant and easily adaptable for future pandemics or health crises.

**Table 3:** Quotes relating to themes arising from the focus groups with clinicians.

Themes	Example quotes
Challenges and uncertainty during the COVID-19 pandemic and beyond	“It’s absolutely decimated us from the start; we’ve had outbreak after outbreak with our patients...it’s been horrific and continues to be as bad as the onset.” [Social worker; male; UK^a^ 1] “In the early days, we did have to really fight the corner for mental health within...the different services and settings that we have. That, kind of, continued I guess throughout the whole vaccine rollout as well.” [Pharmacist; female; UK 3] “I have seen a lot of people [patients] staying stagnant with us through the COVID period and without the opportunity to go through the rehabilitation phases...I think it had a big impact because people felt like they were stuck there for a very long time.” [Consultant psychiatrist; male; UK 1] “The anatomy of our pandemic...was very different...we initially followed an elimination strategy which was quite successful with the first variants up until Omicron...[we] benefitted from the experience you’d had before.” [Consultant psychiatrist; female; NZ^b^]
The need for reliable and trustworthy information	“It was interesting there were a number of places they [patients and the public] were getting misinformation from.” [Consultant psychiatrist; male; AUS^c^] “Dealing with many patients who are sceptical of many things such as worldwide pandemics was very challenging.” [Nurse; male; UK 1] “Our wards and our medical teams but also our patients needed to understand what was a really ever-changing picture.” [Pharmacist; female; UK 3] “To be honest, at the very beginning, I think we went back to almost first principles...At the very, very early stages, that’s what it likely came down to, was independent clinical decision making.” [Pharmacist; male; UK 2] “There were definitely areas that we were grappling with, you know, depots, clozapine, benzos, rapid tranq [sic], and also that whole, sort of, vaccine hesitancy and the confusion that came really with different groupings of who went when.” [Pharmacist; female; UK 3] “A lot of the patients we see do have complicated problems with thyroid, renal function complications, combination strategies, etc., and lithium’s always a difficult molecule to manage in that environment.” [Consultant psychiatrist; male; AUS] “A lot of this evidence is around supporting clinicians to make decisions, not about making them for them, so that if you’re going to take a risk, you’re supporting that risk.” [Pharmacist; male; UK 2]
Feedback about the current OxPPL^d^ guidance	“So, I think it’s really fantastic, and I wish, like, we’d probably had sight of some of this before to help with some issues that we’ll have had in all sorts of our cases, that we’ve had to manage during the whole of the pandemic.” [Nurse matron; female; UK 2] “I think that just the dissemination and reach is where it could be improved. I just think it’s only useful if people are actually reading it and using it.” [Mental health care assistant; female; UK 4] “I like the way that it’s laid out by clinical questions and it feels that it’s had a lot of clinical input, that you’ve thought carefully about what is going to matter to patients, families and clinicians. So, the questions are really good.” [Consultant psychiatrist; female; NZ] “Many colleagues that I’ve forwarded it to recently have found it immensely relevant.” [Social worker; male; UK 1] “So I would see it as...evidence-based, this is authoritative.” [Consultant psychiatrist; male; UK 4] “The clinical relevance is the bit that I loved the most. I had questions and I looked and there they were.” [Consultant psychiatrist; female; NZ] “I did notice there were sections that were relevant to specific professions, like there was nursing and...I always appreciate that, to find things that are specific for what we’re doing.” [Nurse; female; NZ] “The first thing that really struck me was the immense number of topics...and the thoroughness with which they’ve been covered...Also, some of the evidence and data that you’ve drawn on to substantiate various claims, again, it was very nice to see that.” [Consultant psychiatrist; male; AUS] “I remember when I wanted to join the clozapine clinic...I got loads of documents to go through, it was so much. I felt the information we have on the [OxPPL] guidelines, is compact, something that one can go through within a few minutes...before you go in for the clinic. So I think it’s much relevant to this present time that we are in.” [Mental health care assistant; male; UK 2] “So, it’s useful in times of great uncertainty where people don’t know what the right thing to do is and they’re worried about what the risks might be to themselves, for example, about clozapine or lithium treatment.” [Consultant psychiatrist; female; NZ] “We’re not in [a] pandemic now, but they’re still useful.” [Psychiatry trainee; female; UK 2]
Ideas about future use and relevance to future pandemics	“It reinforces what you’ve said and they [patients] do remember it better because when they’re reading it later on and it says the same stuff you’ve just read out with them, it makes a lot more impact, or they share it with their partner or someone else...” [Consultant psychiatrist; male; AUS] “I think personally, over the next two to three years...COVID [will] probably remain a topical issue and a pressure in the winter.” [Pharmacist; male; UK 2] “As a model, I think it’s highly relevant to future pandemics or health crises and you could follow that model quite tightly and come out with something excellent much faster than you could the first time.” [Consultant psychiatrist; female; NZ] “So, I think there is a significant role for this kind of guidance going forward...I think if we are susceptible to one pandemic, maybe we might be susceptible to others, as well.” [Consultant psychiatrist; male; UK 1] “So I think that what you’d need to do is have a critical mass of people who could quickly mobilise to generate those questions and check the specific evidence relating to those questions quickly, because there is quite a bit of intensive resourcing that needs to go in, particularly at the beginning, and then keeping something up to date to make it relevant to the front-of-mind questions that people have.” [Consultant psychiatrist; female; NZ]

^a^UK: United Kingdom; sites are numbered from 1 to 4.

^b^NZ: New Zealand.

^c^AUS: Australia.

^d^OxPPL: Oxford Precision Psychiatry Lab.

### Systematic Review

**Figure 2:**
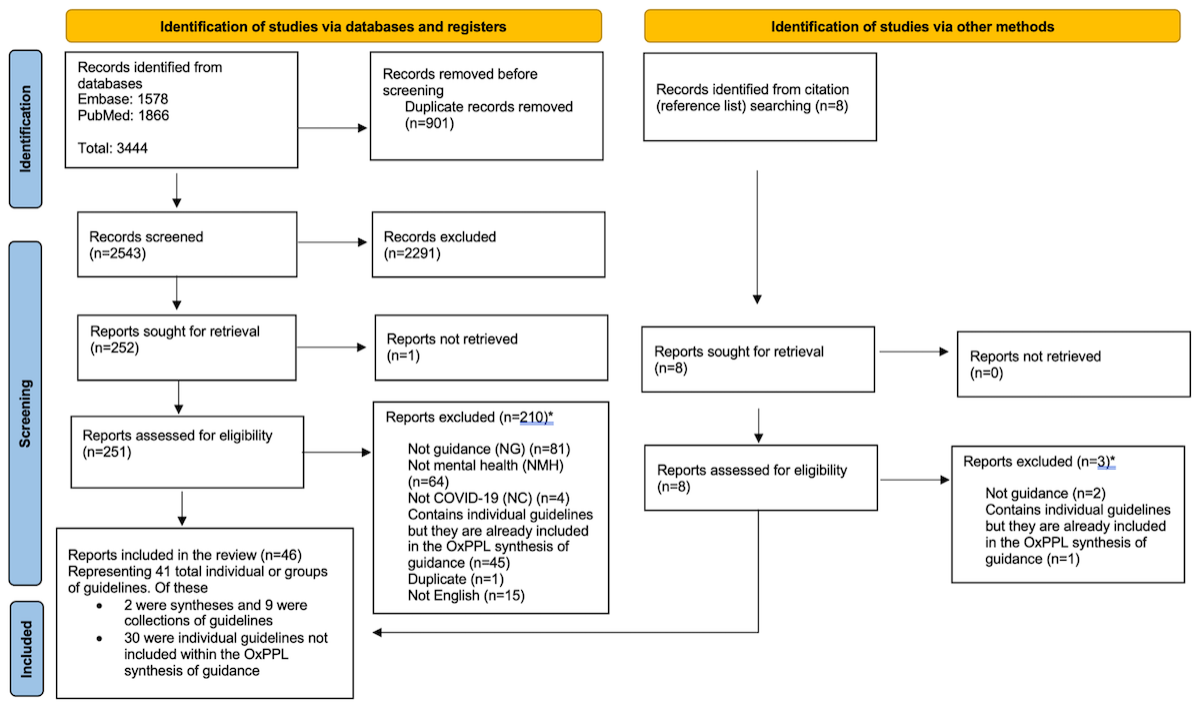
PRISMA (Preferred Reporting Items for Systematic Reviews and Meta-Analyses) flowchart. OxPPL: Oxford Precision Psychiatry Lab; *Single reason is given for exclusion as per the protocol, but many fulfilled multiple exclusion criteria.

The PRISMA flowchart is shown in Figure 2 [28]. We identified a total of 3444 records, which reduced to 2543 records after the removal of duplicates. After screening the abstracts and full texts, a total 46 papers, representing 41 individual guidelines or collections and syntheses of guidelines, were included in the review.

The extracted papers are listed in Tables 4 and 5 [3, 19-21, 31-40, 41, 42] and Multimedia Appendix 7 [43-74]. The papers were divided into three main types:

1. Papers in which the authors or group had aimed to collect and then summarize the currently available guidance on mental health care and COVID-19 (syntheses of guidance;
  Table 4).
2. Papers in which the authors or groups had collected the available guidance on mental health care and COVID-19 without summary or synthesis (“collections” of guidance;
  Table 5).
3. Individual guidelines that would have been eligible for inclusion but had not been included within the OxPPL guidance (Multimedia Appendix 7).

Of the 2 syntheses specifically focusing on mental health disorders, the systematic review identified only 1 record in addition to the OxPPL guidance, which reported the summary guidance from the UK Royal College of Psychiatrists [42]. An additional 9 papers were identified describing collections of guidance. These were narrative reviews of guidelines either for specific areas within mental health care, such as tele–mental health or electroconvulsive therapy, or collections of individual guidelines within a specific geographical area or country (Tables 4 and 5).

In total, 30 individual guidelines were retrieved (Multimedia Appendix 7), which had not been included in the OxPPL guidance. Most (24/30) could be excluded because our guidance did not cover those specific areas or because they were not available on the web, and therefore, our methodology could not have captured them. However, our systematic search revealed 6 guidelines that were eligible and had not been included. Of these 6 guidelines, 4 related to specialist areas within telepsychiatry, 1 to behavioral emergencies, and 1 to inpatient care of adults with cognitive impairment, each within the context of COVID-19.

**Table 4:** Syntheses of guidance about mental health and COVID-19.

Type	Group or organization	Publication	Dates	Methodology	Key aims and web link (if applicable)	Country or language
Synthesis	Royal College of Psychiatrists [41]	Burn and Mudholkar [33] 2020	2020 to June 2022	Expert consensus Developed with input from the COVID-19 college advisory group and approved by the college registrar	Synthesis of guidance regarding mental health in the context of COVID-19 in the United Kingdom [42]	United Kingdom
Synthesis	Oxford Precision Psychiatry Lab [15]	Ostinelli et al [20], 2022; Smith et al [19], 2020; Smith et al [3], 2020; and Smith et al [21], 2023	2020 to 2023	Evidence-based approach Systematic search, reference checking, and extraction	Synthesis of available clinical guidelines regarding mental health in the context of the COVID-19 pandemic Evidence-based approach covering existing guidelines in the English language (covering the United Kingdom, United States, Canada, Australia, New Zealand, and Singapore) and translated for use in 6 other countries (China, Italy, Bulgaria, France, Japan, and Turkey) [24]	United Kingdom, United States, Singapore, Canada, Australia, and New Zealand

**Table 5:** Collections of guidance about mental health and COVID-19.

Type	Group or organization	Publication	Dates	Methodology	Key aims and web link (if applicable)	Country or language
Collection	—^a^	Sugarman and Busch [40] 2023	Up to February 23, 2022	Systematic search of reviews/meta-analyses Narrative review of guidelines	Systematic review of tele–mental health or telepsychiatry reviews, systematic reviews, and meta-analyses Narrative review of the guidelines regarding tele–mental health from several relevant major mental health organizations	English-language resources
Collection	—	Bojdani et al [32] 2020	Published in May 2020	Narrative review combined with informal survey and discussions with colleagues	Narrative review and PubMed search of the guidelines available in the United States for mental health issues related to COVID-19	United States
Collection	—	Rangaswamy et al [37] 2022	Published in October 2022	Narrative review combined with expert opinions from informal sessions, talks, and interviews	Narrative review of Indian mental health care services provided during the COVID-19 pandemic, including the guidelines issued	India
Collection	—	Molebatsi et al [35] 2021	Published in May 2021	Web-based search for relevant health guidelines, with emails to request guidelines from mental health practitioners in various sub-Saharan African countries, and expert opinions from interviews	Narrative summary of sub-Saharan African guidelines regarding mental health and psychological support in response to COVID-19	Sub-Saharan African countries
Collection	—	Choi et al [34] 2020	Published in September 2020	Narrative review of guidelines	Narrative review of recommendations or guidelines for pregnancy and childbirth during the COVID-19 pandemic, supplemented by the authors’ recommendations for clinical practice	English-language resources
Collection	—	Alqahtani et al [31] 2021	2020	Narrative review of guidelines	Narrative review of existing guidelines for telepsychology services to inform and adapt to Saudi Arabia and other Arabic communities	Saudi Arabia
Collection	—	Samy et al [38] 2021	2020 and 2021	Web-based search of PubMed, Web of Science, and Google Scholar	Narrative review of primary studies and guidelines regarding mental health and COVID-19 in the Asia-Pacific region	Asia-Pacific region
Collection	—	Purushothaman et al [36] 2020	2020	Narrative review of research databases and existing guidelines	Narrative review of guidelines regarding anesthesia during ECT^b^ and its status within aerosol-generating procedures, supplemented by the authors’ recommendations about ECT and use of PPEc	English-language resources
Collection	Individual members of the Network of Early Career Professionals working in Addiction Medicine	Scheibein et al [39] 2020	6 months following the COVID-19 pandemic	Narrative review of guidelines	Narrative review of country-level guidelines regarding addiction medicine developed in the 6 months following the COVID-19 pandemic	Worldwide

^a^Not reported.

^b^ECT: electroconvulsive therapy.

^c^PPE: personal protective equipment.

## Discussion

### Principal Findings

In this study, we established a multicenter, international network of sites to assess the real-world clinical implementation of web-based, evidence-based, mental health guidance resources in the context of COVID-19. We reviewed the available evidence about approaches to providing guidance for clinicians in mental health during global health emergencies, using the COVID-19 pandemic as an example. We evaluated clinicians’ attitudes toward the need for guidance in general and the OxPPL guidance in particular. We assessed their views about the usefulness of such an approach in future health crises or seasonal demands on health care services and sought to identify the elements needed in preparation for future challenges.

Through the survey and focus groups, clinicians reported that (1) there was a clinical, unmet need for easy-to-access summaries of evidence-based guidance in mental health care during the pandemic, and this need was likely to continue afterward; (2) the web-based, evidence-based summaries of guidance (OxPPL guidance) were clinically useful; (3) they would have or had an impact on their clinical practice; (4) the combination of evidence-based knowledge and a clinical viewpoint was crucial for clinical implementation; and (5) the methodology and web-based format were relevant to future use, including for seasonal surges in illness, future pandemics, and patient adaptation. However, the evaluation also showed that a significant number of the clinicians were not aware of the OxPPL guidance.

### Comparison With Previous Studies

Our results are broadly consistent with those of a previous study. Millard et al [75] used surveys and a small number of individual interviews with Australian clinicians to assess attitudes toward the Australian National COVID-19 Clinical Evidence Taskforce living guidelines [76]. Consistent with our findings, they reported that the guidelines were assessed as being relevant to their practice and trustworthy. More than 50% of the respondents had used the guidelines to support their own clinical decision-making (however, they were not explicitly asked whether the guidelines had made a significant impact). They also found that frequent updates and an evidence-based and clinician-led approach were key qualities. However, in contrast to the OxPPL guidance, this focused on 1 country and on the general medical rather than mental health care context.

During a fast-moving pandemic, frequent and responsive updates are important. Guidelines are usually based on evidence from systematic reviews, but these take time and resources to produce, and in the context of an evolving pandemic, they can quickly become out of date. Living systematic reviews can be used to inform clinical guidelines in a more adaptive manner. Examples include the Australian National COVID-19 Clinical Evidence Taskforce living guidelines [76,77]; the World Health Organization’s Therapeutics and COVID-19: Living Guidelines [78]; and the Global Alliance for Living Evidence on Anxiety, Depression, and Psychosis (GALENOS) [79,80]. However, these guidelines also require a significant investment of time and resources to maintain, and therefore, a pragmatic, evidence-based approach, such as that used in the OxPPL guidance, may be an alternative.

The need for adaptability and responsiveness to a changing environment during the COVID-19 pandemic were key themes raised by clinicians during the focus groups and will be important aspects of preparing the response to future health emergencies. The COVID-19 pandemic resulted in a rapid transition in clinical practice from in-person consultation to telepsychiatry [17], and clinicians in the focus groups described how they were required to quickly adapt their skills to remote consultations. However, a study of mental health professionals during the COVID-19 pandemic suggests that not all clinicians were able to adapt easily and that lower self-ratings of digital competence were associated with higher rates of stress [81]. Realizing the full potential of digital interventions to increase the access to and quality of mental health care both in the aftermath of COVID-19 and in planning for other subsequent health emergencies will require clinicians to feel confident and competent in integrating both synchronous approaches (such as telephone or video consultations) and asynchronous techniques (such as the use of apps or smartphones for monitoring and delivering treatments) into the clinical setting [82]. Teaching and training in telepsychiatry and in digital mental health will be essential elements in planning for the next crises [83]. Although there are many potential challenges in implementing training in digital mental health, the first step will be providing easily accessible summaries of evidence-based knowledge, such as the OxPPL guidance, to extend the skills and competencies of clinicians. Training for patients and carers will also be equally important to allow them to access the best combination of evidence and treatments that are available [82].

For successful real-world implementation, the information provided in guidelines also needs to be effectively integrated into clinical decision-making [9]. Guidelines should be freely accessible, with the methodology, potential conflicts of interest, and dates of the updates clearly defined, so that clinicians can assess the reliability of the recommendations. In addition, uptake in the clinic also depends on the willingness of both clinicians and patients to change and the capacity of clinicians to keep up with new recommendations and provide the additional clinician time needed [84]. However, current frameworks for producing guidelines do not yet formally assess patient or clinician burden [84,85] or context [7] in their implementation. Nonadherence to guidelines has important consequences and can contribute to increased adverse outcomes such as hospitalizations, mortality rates, and health care spending [86]. COVID-19 was a recent example where the real-world impact of rapid, evidence-based guidelines could be assessed. This study provides an assessment of the impact and clinical utility of the OxPPL guidance: guidelines will only make an impact on patient care if they are acted upon by clinical teams, and therefore, any approach to guideline development needs to be formally assessed in terms of its impact in real-world clinical care.

Visibility of the resources was a challenge. The results of the survey and focus groups emphasized the need to combine key elements: evidence-based methodology; clinical relevance; and providing the resources on a recognized platform, which would be quickly accessible and adaptable for future health crises. The systematic review identified only 1 other similar resource. Although this was on a well-recognized platform provided by the UK Royal College of Psychiatrists [42], the guidance was not synthesized using evidence-based methods and was predominantly focused on the United Kingdom.

### Strengths and Limitations

We recognize that this study has some limitations. Survey response rates were very low, despite active reminders. These are perhaps explained by a level of COVID-19 fatigue or burnout [87,88] at the time of the survey. In addition, each site used their existing email databases for staff. At least some of these databases had significant overlaps with non–mental health staff and non–patient-facing staff (such as administration, or technology support staff). Although the email and survey specified that the survey applied only to patient-facing mental health care staff and that staff could not proceed further if they were not involved in mental health care and were not patient facing, these factors mean that the response rate may be inaccurate and appear to be significantly lower because of an overestimate of the denominator.

Although the survey was launched across multiple sites, all 4 sites in the United Kingdom were in England, and of the Australian sites, Sydney participated only in the focus group and Brisbane participated only in the survey. In addition, our evaluation was undertaken only in English-speaking countries, as adding other languages would have complicated the analysis of the survey and focus groups. However, the survey provided a wide context with a multidisciplinary and international perspective across sites in England, Australia, and New Zealand. Both the survey and focus group feedback was subjective and reflected only the views of mental health care staff about the resources that had been developed for use primarily by clinicians. Several clinicians highlighted how they had also used the guidance usefully in their clinical interactions with patients, and there were many suggestions regarding modifications for patient use. However, further studies would be needed to explore the possible adaptations of the resources using feedback from patients and carers.

The survey and focus group findings highlighted how strongly clinicians felt that reliable guidance in mental health care was needed during health crises. Overall, 80.2% (146/182) of those surveyed felt that the methods and approaches used in the OxPPL guidance would be helpful in addressing the need for mental health care information during future pandemics or health crises. The advantage of our international focus was that it included perspectives from different countries, with focus group participants from Australia and New Zealand highlighting their different pandemic-related experiences. The COVID-19 elimination strategy in New Zealand and stringent lockdowns in Australia meant that case rates were often lower, whereas rates were higher in other countries. During these times, participants reported looking for guidance from countries such as the United Kingdom, the United States, and Canada that had higher rates of COVID-19 but struggled to find reliable, easily accessible sources of guidance in mental health care, particularly during the early part of the pandemic.

We actively updated the resources according to the stage of the pandemic but used a pragmatic, evidence-based approach: rather than a systematic review of the published literature, we used a modified method in which we rapidly and systematically searched web-based sources of guidance [3]. When we performed a systematic review, across all the mental health areas covered in the OxPPL guidance we found only 6 individual guidelines that would have been eligible but were not included. Although this number is low, it may indicate some potential limitations to our approach; the advantages of speed and responsiveness mean that guidelines, particularly those hosted on specialized or region-wide rather than country-wide websites, could be missed. When reviewing the omitted guidelines, we found that 5 of the 6 guidelines were in more specialized resources that we had not searched, which supports this hypothesis. Given the huge array of available guidelines produced during the COVID-19 pandemic, it is also possible that our systematic review could also have missed some reports.

### Conclusions

In summary, we have suggested a successful, evidence-based approach during the pandemic, and our formal assessment of its impact supports the usefulness and relevance of resources such as these in real-world, clinical implementation in mental health care. Now is the time to prepare for the next challenge. COVID-19 is the most recent and striking example of a global health pandemic, but it is by no means the only one. There have been numerous other outbreaks of infectious diseases in the past century, including influenza pandemics or epidemics and severe outbreaks of Ebola and of other coronaviruses [89], and there will be further health crises in the future. The need for timely evidence and rapid and effective communication of recommendations to clinicians to respond to global health emergencies such as COVID-19 is currently at the front of our minds, and this is the ideal time to review the quality of our response and identify learning points [90,91]. Preparing for the next health crisis will also include addressing the training needs of clinicians, patients, and carers, especially in areas such as telepsychiatry and digital mental health [83,92].

The combination of evidence-based knowledge and clinical expertise in mental health care is needed, but this can only be truly successful if hosted on an easily accessible, widely disseminated, web-based platform, and digital technology can materially help [92]. Creating a living document with responsive and frequent updates demands significant resources and a dedicated approach from an international and multidisciplinary team. Establishing all these things now would ensure effective and prompt synthesis of guidance when it is needed in the future [93]. Governments and funding agencies across the world should be aware of this and start preparing immediately to be ready when the next pandemic or international health emergency arises [94].

## Appendix

Multimedia Appendix 1 Focus group topic guide.

Multimedia Appendix 2 PRISMA (Preferred Reporting Items for Systematic Reviews and Meta-Analyses) checklist.

Multimedia Appendix 3 Characteristics of respondents to the survey.

Multimedia Appendix 4 Survey responses.

Multimedia Appendix 5 Characteristics of focus group participants.

Multimedia Appendix 6 Framework analysis of the focus group transcripts.

Multimedia Appendix 7 Individual guidelines about mental health and COVID-19, which were not originally included in the Oxford Precision Psychiatry Lab guidance.

## Abbreviations

COREQ: Consolidated Criteria for Reporting Qualitative Research
GALENOS: Global Alliance for Living Evidence on Anxiety, Depression, and Psychosis
NHS: National Health Service
OxPPL: Oxford Precision Psychiatry Lab
PRISMA: Preferred Reporting Items for Systematic Reviews and Meta-Analyses
