# Supplementary material for: Assessing the Impact of Evidence-Based Mental Health Guidance During the COVID-19 Pandemic: Systematic Review and Qualitative Evaluation
Source: JMIR Ment Health. 2023 Dec 22;10:e52901. doi: 10.2196/52901 (PMC10760515; doi:10.2196/52901)
Supplement: Multimedia Appendix 1 [file mental_v10i1e52901_app1.docx]

**Multimedia appendix 1: Focus group topic guide**

The following questions are intended as guidance questions for the focus group and will be developed as the focus group progresses.

**Openers**

Can you describe the clinical settings you work in? (include country, setting, professional background)

Can you say a little bit about your work role during the COVID-19 pandemic and afterwards?

In your experience, where did you go to for information about mental health care delivery for your patients during the pandemic?

**Follow on questions**

Were you aware of the OxPPL guidance before this study? If so, how did you hear about it?

Now you have had a chance to look at it, what do you think are the positives/good things about it?

- Is it clinically relevant? Why/why not?
- Easy to use? Why/why not?
- Trustworthy/reliable in terms of methods? Why/why not?
- Extra features? (As well as the webpages with embedded hyperlinks, there were also downloadable PDFs to print out the summaries and a Word version with all the original links for those who wished more detail) Why/why not?

What are the negatives/ what could be improved about the OxPPL guidance?

Which topics did you look at/like the most? What were the reasons for this?

What changes would you like to see?

- In layout, content and format?
- In terms of topics covered – can you suggest others we should cover?

Do you feel that the OxPPL guidance is more relevant to certain professional groups than others?

Would you share it with others? If so who (colleagues/patients/carers?) and why?

**Planning for the future**

Is the OxPPL guidance a helpful model for providing information in future crises or only applicable to the COVID-19 pandemic? Why is this?

Are there any adaptations that would need making to the OxPPL guidance to ensure it is relevant and applicable in future pandemics/health crises?
