# Supplementary material for: Assessing the Impact of Evidence-Based Mental Health Guidance During the COVID-19 Pandemic: Systematic Review and Qualitative Evaluation
Source: JMIR Ment Health. 2023 Dec 22;10:e52901. doi: 10.2196/52901 (PMC10760515; doi:10.2196/52901)
Supplement: Multimedia Appendix 3 [file mental_v10i1e52901_app3.docx]

**Multimedia appendix 3:** **Characteristics of respondents to the survey**

|  | UK (n = 120) | New Zealand (n = 48) | Australia  (n = 16) | Total  (n = 184) | |
| --- | --- | --- | --- | --- | --- |
| **Professional background** |  |  |  |  | |
| Medical | 28 (23%) | 8 (17%) | 1 (6%) | 37 (20%) | |
| Nursing | 46 (38%) | 24 (50%) | 4 (25%) | 74 (40%) | |
| Occupational therapy | 1 (1%) | 3 (6%) | 1 (6%) | 5 (3%) | |
| Pharmacy | 6 (5%) | 0 (0%) | 3 (19%) | 9 (5%) | |
| Physiotherapy | 2 (2%) | 0 (0%) | 0 (0%) | 2 (1%) | |
| Psychology | 14 (12%) | 9 (19%) | 2 (13%) | 25 (14%) | |
| Social work | 10 (8%) | 2 (4%) | 5 (31%) | 17 (9%) | |
| Other | 13 (11%) | 2 (4%) | 0 (0%) | 15 (8%) | |
| **Prescribing healthcare worker** |  |  |  |  | |
| Yes | 31 (26%) | 10 (21%) | 1 (6%) | 42 (23%) | |
| No | 89 (74%) | 38 (79%) | 15 (94%) | 142 (77%) | |
| **Main service (more than 50% of the time)** |  |  |  |  | |
| Adult mental health | 65 (54%) | 18 (38%) | 13 (81%) | 96 (52%) | |
| Child and adolescent mental health | 9 (8%) | 12 (25%) | 1 (6%) | 22 (12%) | |
| Forensic mental health | 12 (10%) | 13 (27%) | 1 (6%) | 26 (14%) | |
| Learning disability | 6 (5%) | 0 (0%) | 0 (0%) | 6 (3%) | |
| Mental health long-term rehabilitation | 0 (0%) | 0 (0%) | 0 (0%) | 0 | |
| Memory assessment clinic | 3 (3%) | 0 (0%) | 0 (0%) | 3 (2%) | |
| Older adult mental health | 7 (6%) | 2 (4%) | 1 (6%) | 10 (5%) | |
| Not mental health-related | 2 (2%) | 0 (0%) | 0 (0%) | 2 (1%) | |
| Other | 16 (13%) | 3 (6%) | 0 (0%) | 19 (10%) | |
| **Health service** |  |  |  |  | |
| Berkshire Health | 2 |  |  |  | |
| Hertfordshire NHS FT | 1 |  |  |  | |
| Lincolnshire NHS FT | 31 |  |  |  | |
| Mersey Care NHS FT | 13 |  |  |  | |
| Oxford University Hospital NHS FT | 1 |  |  |  | |
| Oxford Health NHS FT | 63 |  |  |  | |
| Pennine NHS FT | 7 |  |  |  | |
| Queensland Health |  |  | 15 |  | |
| Royal Brisbane and Women’s Hospital |  | | 1 | |  |
| Sydney |  |  | 0 |  | |
| NA | 2 |  |  |  | |
| **Main work setting** |  |  |  |  | |
| Inpatient | 36 | 21 | 7 | 64 | |
| Outpatient | 24 | 4 | 2 | 30 | |
| Community team | 43 | 16 | 6 | 65 | |
| Other | 15 | 7 | 1 | 23 | |
| NA | 2 | 0 | 0 | 2 | |
| **Age** |  |  |  |  | |
| 18-24 | 9 | 0 | 0 | 9 (5%) | |
| 25-34 | 29 | 14 | 3 | 46 (25%) | |
| 35-44 | 24 | 11 | 6 | 41 (22%) | |
| 45-54 | 34 | 10 | 3 | 47 (26%) | |
| 55-64 | 16 | 10 | 4 | 30 (16%) | |
| 65-74 | 5 | 0 | 0 | 5 (3%) | |
| 75+ | 0 | 2 | 0 | 2 (1%) | |
| Prefer not to say | 1 | 1 | 0 | 2 (1%) | |
| NA | 2 | 0 | 0 | 2 (1%) | |
| **Gender** |  |  |  |  | |
| Female | 78 | 31 | 11 | 120 (65%) | |
| Male | 39 | 16 | 5 | 60 (33%) | |
| Non-binary | 1 | 0 | 0 | 1 (0.5%) | |
| Prefer not to say | 0 | 1 | 0 | 1 (0.5%) | |
| NA | 2 | 0 | 0 | 2 91%) | |
| **Ethnicity** |  |  |  |  | |
| New Zealand European |  | 25 (52%) |  |  | |
| Māori |  | 1 (2%) |  |  | |
| Māori – New Zealand European |  | 6 (13%) |  |  | |
| Samoan |  | 0 |  |  | |
| Cook Island Māori |  | 0 |  |  | |
| Cook Island Māori – Māori – Tokelauan |  | 1 |  |  | |
| Tongan |  | 0 |  |  | |
| Niuean |  | 0 |  |  | |
| Indian |  | 2 (4%) |  |  | |
|  |  |  |  |  | |
| Asian or Asian British background | 8 (7%) |  |  |  | |
| *Bangladeshi* | *0* |  |  |  | |
| *Indian* | *5* |  |  |  | |
| *Pakistani* | *1* |  |  |  | |
| *Any other* | *2* |  |  |  | |
| Black or black British | 4 (3%) |  |  |  | |
| *African* | *4* |  |  |  | |
| *Caribbean* | *0* |  |  |  | |
| *Any other* | *0* |  |  |  | |
| Chinese | 0 | 2 (4%) | 0 |  | |
| White background | 97 (81%) | 2 (4%) | 2 (13%) |  | |
| *British* | *85* | *1* |  |  | |
| *Irish* | *2* |  |  |  | |
| *Any other* | *10* | *1* | *2* |  | |
| Mixed | 5 (4%) |  |  |  | |
| *White and*  *Black Caribbean* | *0* |  |  |  | |
| *White and Black African* | *2* |  |  |  | |
| *White and Asian* | *2* |  |  |  | |
| *Any other* | *1* |  |  |  | |
|  |  |  |  |  | |
| Oceanian |  |  | 7 (44%) |  | |
| *Australian people* |  |  | *6* |  | |
| *New Zealand* |  |  | *1* |  | |
| *Melanesian and Papuan* |  |  | *0* |  | |
| *Micronesian* |  |  | *0* |  | |
| *Polynesian* |  |  | *0* |  | |
| Northern and western European |  |  | 2 (13%) |  | |
| Southern and eastern European |  |  | 0 |  | |
| Northern African and middle eastern |  |  | 0 |  | |
| Sub-Saharan African |  |  | 0 |  | |
| Northern and eastern Asian |  |  | 3 (19%) |  | |
| Southern and central Asian |  |  | 0 |  | |
| Southern and eastern Asian |  |  | 1 (6%) |  | |
| People of the Americas |  |  | 0 |  | |
| Prefer not to say | 3 (3%) | 2 (4%) | 1 (6%) |  | |
| Other | 1 (1%) | 7 (15%) | 0 |  | |
| NA | 2 (2%) | 0 | 0 |  | |
