# Supplementary material for: Assessing the Impact of Evidence-Based Mental Health Guidance During the COVID-19 Pandemic: Systematic Review and Qualitative Evaluation
Source: JMIR Ment Health. 2023 Dec 22;10:e52901. doi: 10.2196/52901 (PMC10760515; doi:10.2196/52901)
Supplement: Multimedia Appendix 4 [file mental_v10i1e52901_app4.docx]

**Multimedia appendix 4:** **Survey responses**

| **Before completing this questionnaire, had you read or used the OxPPL guidance?** | | | | |
| --- | --- | --- | --- | --- |
| Yes | 58 (48%) | 17 (35%) | 4 (25%) | 79 (43%) |
| No | 60 (50%) | 31 (65%) | 12 (75%) | 103 (56%) |
| NA | 2 (2%) | 0 (0%) | 0 (0%) | 2 (1%) |

Users familiar with the OxPPL guidance:

| Question | UK (n = 58) | NZ (n = 17) | AU (n = 4) | Total (n = 79) |
| --- | --- | --- | --- | --- |
| Benzodiazepines and Z-drugs | Not used: 41  < 2 times: 7  2 to 3 times: 4  4 to 6 times: 3  > 6 times: 3 | Not used: 16  < 2 times: 1  2 to 3 times: 0  4 to 6 times: 0  > 6 times: 0 | Not used: 2  < 2 times: 2  2 to 3 times: 0  4 to 6 times: 0  > 6 times: 0 | Not used: 59  < 2 times: 10  2 to 3 times: 4  4 to 6 times: 3  > 6 times: 3  (Used by 25% overall) |
| Clozapine treatment | Not used: 41  < 2 times: 6  2 to 3 times: 7  4 to 6 times: 0  > 6 times: 4 | Not used: 15  < 2 times: 0  2 to 3 times: 0  4 to 6 times: 0  > 6 times: 2 | Not used: 3  < 2 times: 1  2 to 3 times: 0  4 to 6 times: 0  > 6 times: 0 | Not used: 59  < 2 times: 7  2 to 3 times: 7  4 to 6 times: 0  > 6 times: 6  (Used by 25% overall) |
| Digital technologies and telepsychiatry | Not used: 36  < 2 times: 9  2 to 3 times: 4  4 to 6 times: 3  > 6 times: 6 | Not used: 11  < 2 times: 4  2 to 3 times: 1  4 to 6 times: 0  > 6 times: 1 | Not used: 4  < 2 times: 0  2 to 3 times: 0  4 to 6 times: 0  > 6 times: 0 | Not used: 51  < 2 times: 13  2 to 3 times: 5  4 to 6 times: 3  > 6 times: 7  (Used by 35% overall) |
| Domestic violence and abuse | Not used: 38  < 2 times: 9  2 to 3 times: 4  4 to 6 times: 4  > 6 times: 3 | Not used: 14  < 2 times: 3  2 to 3 times: 0  4 to 6 times: 0  > 6 times: 0 | Not used: 4  < 2 times: 0  2 to 3 times: 0  4 to 6 times: 0  > 6 times: 0 | Not used: 56  < 2 times: 12  2 to 3 times: 4  4 to 6 times: 4  > 6 times: 3  (Used by 29% overall) |
| End of life care | Not used: 51  < 2 times: 4  2 to 3 times: 2  4 to 6 times: 1  > 6 times: 0 | Not used: 17  < 2 times: 0  2 to 3 times: 0  4 to 6 times: 0  > 6 times: 0 | Not used: 4  < 2 times: 0  2 to 3 times: 0  4 to 6 times: 0  > 6 times: 0 | Not used: 72  < 2 times: 4  2 to 3 times: 2  4 to 6 times: 1  > 6 times: 0  (Used by 1% overall) |
| Inpatients | Not used: 39  < 2 times: 3  2 to 3 times: 7  4 to 6 times: 2  > 6 times: 7 | Not used: 8  < 2 times: 6  2 to 3 times: 2  4 to 6 times: 0  > 6 times: 1 | Not used: 3  < 2 times: 1  2 to 3 times: 0  4 to 6 times: 0  > 6 times: 0 | Not used: 50  < 2 times: 10  2 to 3 times: 9  4 to 6 times: 2  > 6 times: 8  (Used by 37% overall) |
| Lithium treatment | Not used: 39  < 2 times: 7  2 to 3 times: 10  4 to 6 times: 1  > 6 times: 1 | Not used: 16  < 2 times: 0  2 to 3 times: 0  4 to 6 times: 0  > 6 times: 1 | Not used: 3  < 2 times: 1  2 to 3 times: 0  4 to 6 times: 0  > 6 times: 0 | Not used: 58  < 2 times: 8  2 to 3 times: 10  4 to 6 times: 1  > 6 times: 2  (Used by 27% overall) |
| Long-acting injectable (LAI) antipsychotics | Not used: 45  < 2 times: 7  2 to 3 times: 4  4 to 6 times: 0  > 6 times: 2 | Not used: 15  < 2 times: 0  2 to 3 times: 2  4 to 6 times: 0  > 6 times: 0 | Not used: 2  < 2 times: 2  2 to 3 times: 0  4 to 6 times: 0  > 6 times: 0 | Not used: 62  < 2 times: 9  2 to 3 times: 6  4 to 6 times: 0  > 6 times: 2  (Used by 22% overall) |
| Pregnancy and the perinatal period | Not used: 47  < 2 times: 6  2 to 3 times: 4  4 to 6 times: 0  > 6 times: 1 | Not used: 15  < 2 times: 1  2 to 3 times: 1  4 to 6 times: 0  > 6 times: 0 | Not used: 4  < 2 times: 0  2 to 3 times: 0  4 to 6 times: 0  > 6 times: 0 | Not used: 66  < 2 times: 7  2 to 3 times: 5  4 to 6 times: 0  > 6 times: 1  (Used by 17% overall) |
| Suicide and self-harm | Not used: 24  < 2 times: 17  2 to 3 times: 4  4 to 6 times: 2  > 6 times: 11 | Not used: 13  < 2 times: 2  2 to 3 times: 1  4 to 6 times: 1  > 6 times: 0 | Not used: 4  < 2 times: 0  2 to 3 times: 0  4 to 6 times: 0  > 6 times: 0 | Not used: 41  < 2 times: 19  2 to 3 times: 5  4 to 6 times: 3  > 6 times: 11  (Used by 48% overall) |
| Vaccine prioritisation and mental health | Not used: 35  < 2 times: 7  2 to 3 times: 6  4 to 6 times: 5  > 6 times: 5 | Not used: 12  < 2 times: 2  2 to 3 times: 1  4 to 6 times: 1  > 6 times: 1 | Not used: 4  < 2 times: 0  2 to 3 times: 0  4 to 6 times: 0  > 6 times: 0 | Not used: 51  < 2 times: 9  2 to 3 times: 7  4 to 6 times: 6  > 6 times: 6  (Used by 35% overall) |
| Vaccine uptake and vaccine hesitancy | Not used: 29  < 2 times: 15  2 to 3 times: 4  4 to 6 times: 3  > 6 times: 7 | Not used: 12  < 2 times: 3  2 to 3 times: 1  4 to 6 times: 1  > 6 times: 0 | Not used: 4  < 2 times: 0  2 to 3 times: 0  4 to 6 times: 0  > 6 times: 0 | Not used: 45  < 2 times: 18  2 to 3 times: 5  4 to 6 times: 4  > 6 times: 7  (Used by 43% overall) |

Users not familiar with the OxPPL guidance:

| **Which guidance did you look at?** *Multiple choice question. Percentages refer to the country-specific total figure.* | | | | |
| --- | --- | --- | --- | --- |
| Guidance | UK (n = 60) | NZ (n = 31) | AU (n = 12) | Total (n = 103) |
| Benzodiazepines and Z-drugs | 19 (32%) | 8 (26%) | 2 (17%) | 29 (28%) |
| Clozapine treatment | 18 (30%) | 13 (42%) | 4 (33%) | 35 (34%) |
| Digital technologies and telepsychiatry | 16 (27%) | 8 (26%) | 0 (0%) | 24 (23%) |
| Domestic violence and abuse | 10 (17%) | 5 (16%) | 4 (33%) | 19 (18%) |
| End of life care | 8 (13%) | 1 (3%) | 1 (8%) | 10 (10%) |
| Inpatients | 8 (13%) | 10 (32%) | 2 (17%) | 20 (19%) |
| Lithium treatment | 8 (13%) | 5 (16%) | 0 (0%) | 13 (13%) |
| Long-acting injectable (LAI) antipsychotics | 6 (10%) | 3 (10%) | 1 (8%) | 10 (10%) |
| Pregnancy and the perinatal period | 2 (3%) | 3 (10%) | 1 (8%) | 6 (6%) |
| Suicide and self-harm | 19 (32%) | 14 (45%) | 5 (42%) | 38 (37%) |
| Vaccine prioritisation and mental health | 6 (10%) | 4 (13%) | 3 (25%) | 13 (13%) |
| Vaccine uptake and vaccine hesitancy | 7 (12%) | 3 (10%) | 1 (8%) | 11 (11%) |

Likert scales

| Question | Strongly disagree | Somewhat disagree | Neither agree or disagree | Somewhat agree | Strongly agree | Total |
| --- | --- | --- | --- | --- | --- | --- |
| The OxPPL guidance answers important clinical questions. | 3 (2%) | 7 (4%) | 26 (14%) | 81 (45%) | 65 (36%) | 182 (100%) |
| The methods used to collect and synthesise the OxPPL guidance are appropriate (i.e. they are trustworthy enough to use in clinical practice). | 1 (1%) | 4 (2%) | 38 (21%) | 73 (40%) | 66 (36%) | 182 (100%) |
| The layout is easy to access and I can find the answers I need. | 2 (1%) | 10 (5%) | 28 (15%) | 68 (37%) | 74 (41%) | 182 (100%) |
| The extra features (e.g. downloadable pdf summary, appendix with full list of original sources) are useful. | 2 (1%) | 5 (3%) | 30 (16%) | 64 (35%) | 81 (45%) | 182 (100%) |
| The OxPPL guidance has had/will have a positive impact on my clinical practice. | 3 (2%) | 10 (5%) | 61 (34%) | 73 (40%) | 35 (19%) | 182 (100%) |
| The OxPPL guidance is relevant and applicable where I work (in terms of type of service). | 3 (2%) | 10 (5%) | 36 (20%) | 67 (37%) | 66 (36%) | 182 (100%) |
| The OxPPL guidance is relevant and applicable where I work (in terms of type of patient population). | 3 (2%) | 13 (7%) | 35 (19%) | 67 (37%) | 64 (35%) | 182 (100%) |

Sharing the OxPPL guidance

| **Have you shared/will share the OxPPL guidance with other people?** | |
| --- | --- |
| Yes | 78 (43%) |
| No | 104 (57%) |
| **Please indicate with whom you shared/will you share the summaries?**  *Multiple choice question. Percentages refer to n = 78.* | |
| Co-workers | 70 (90%) |
| Other professional | 26 (33%) |
| Patient | 16 (21%) |
| Carers | 9 (12%) |
| Other | 4 (5%) |
| **How did/will you share this?** *Multiple choice question. Percentages refer to n = 78.* | |
| Informally/during a conversation | 59 (76%) |
| During a talk/meeting/webinar | 22 (28%) |
| In a paper/publication | 6 (8%) |
| During a clinical consultation | 8 (10%) |
| Other | 10 (13%) |

Future use

| Question | Strongly disagree | Somewhat disagree | Neither agree or disagree | Somewhat agree | Strongly agree | Total |
| --- | --- | --- | --- | --- | --- | --- |
| The methods used here to synthesise and provide summaries of guidance on managing mental health issues could be used in future pandemics/health crises. | 1 (1%) | 3 (2%) | 32 (18%) | 76 (42%) | 70 (38%) | 182 (100%) |
| Additional topics should be included to deal with/manage the needs of people with mental health difficulties during future pandemics. | 3 (2%) | 6 (3%) | 56 (31%) | 62 (34%) | 55 (30%) | 182 (100%) |

| **Which other resources have you used most for guidance in mental health diagnosis, treatment and other services in the context of COVID-19?** *Multiple choice question. Percentages refer to n = 182.* | |
| --- | --- |
| Specialty based website | 26 (14%) |
| Based on professional background | 31 (17%) |
| Governmental websites | 62 (34%) |
| Other | 14 (8%) |
| None | 49 (27%) |
