# Supplementary material for: Assessing the Impact of Evidence-Based Mental Health Guidance During the COVID-19 Pandemic: Systematic Review and Qualitative Evaluation
Source: JMIR Ment Health. 2023 Dec 22;10:e52901. doi: 10.2196/52901 (PMC10760515; doi:10.2196/52901)
Supplement: Multimedia Appendix 5 [file mental_v10i1e52901_app5.docx]

**Multimedia appendix 5:** **Characteristics of focus group participants**

| Professional background | Specialty | Description | Gender | Country |
| --- | --- | --- | --- | --- |
| Doctor | Older adult | Trainee (ST5*) | F | UK (Lincolnshire) |
| Pharmacist | All | Service manager for pharmacy | M | UK (Lincolnshire) |
| Mental Healthcare Assistant | General adult |  | M | UK (Lincolnshire) |
| Nurse | General adult | Modern matron | F | UK (Lincolnshire) |
| Doctor | Adult mental health and low secure | Consultant | M | UK (Mersey Care) |
| Social Work | Forensic |  | M | UK (Mersey Care) |
| Nurse | Forensic |  | M | UK (Mersey Care) |
| Pharmacist | All | Lead pharmacist | F | UK (Pennine) |
| Doctor | Older adult | Trainee (ST5*) | F | UK (Oxford Health) |
| Doctor | General adult | Consultant | M | UK (Oxford Health) |
| Mental Healthcare Assistant | Eating disorders |  | F | UK (Oxford Health) |
| Doctor | Older Adult | Consultant | M | UK (Oxford Health) |
| Doctor | Bipolar disorders/general adult | Consultant | M | Australia (Sydney) |
| Doctor | Forensic | Consultant | F | New Zealand |
| Occupational Therapist | Forensic |  | F | New Zealand |
| Doctor | Forensic | Consultant | M | New Zealand |
| Doctor | Child and Adolescent | Consultant | M | New Zealand |
| Nurse | Forensic |  | F | New Zealand |

*ST5 = Doctor specialty trainee in year 5 of training.
