# Supplementary material for: Assessing the Impact of Evidence-Based Mental Health Guidance During the COVID-19 Pandemic: Systematic Review and Qualitative Evaluation
Source: JMIR Ment Health. 2023 Dec 22;10:e52901. doi: 10.2196/52901 (PMC10760515; doi:10.2196/52901)
Supplement: Multimedia Appendix 6 [file mental_v10i1e52901_app6.docx]

**Multimedia appendix 6: Framework analysis of the focus group transcripts**

UK sites are numbered 1-4, the Australian site as AUS and the New Zealand site as NZ.

**THEME 1: CHALLENGES AND UNCERTAINTY DURING THE COVID-19 PANDEMIC**

Participants used the group to reflect on their experiences during COVID-19, and most noted how they felt that the challenges experienced had changed as the pandemic developed:

*‘My experience was that during the initial periods of COVID everyone was quite scared about almost surviving or physical health is the main thing. As we passed through that big hurdle, ….. it had a toll on mental health.’* [Consultant Psychiatrist, male, UK 1]

Whilst UK-based participants reflected on the impact of lockdowns and waves of infection, participants from New Zealand and Australia noted key differences relating to their experiences, including in New Zealand, an initial period of pursuing an elimination strategy. This impacted on how clinicians and patients approached mental health in New Zealand, with many stressors resulting from taking preventative action, rather than dealing with the virus itself.

*‘The anatomy of our pandemic ….. was very different, …… we initially followed an elimination strategy…….. So our experiences were working in situations with high COVID anxiety and very stringent lockdowns and a lot of stress within clinicians and patients as to how to manage leave restrictions and family visits and all those things in the context of the lockdowns, but not a lot of COVID. Then we had a very big wave of COVID that has passed through this year [2022] with the Omicron variant……….[we] benefitted from the experience you’d had before.*’ [Consultant Psychiatrist, female, NZ]

The majority of participants commented on their experiences of high workload and staff shortages during the pandemic and thereafter:

*‘It's absolutely decimated us from the start; we've had outbreak after outbreak with our patients. With the staffing in [city], it's been horrific and continues to be as bad as the onset.’* [Social worker, male, UK 1]

Several participants commented on the perceived pressure and urgency to implement changes during the pandemic. For example, a consultant psychiatrist commented that in terms of telepsychiatry, remote assessments quickly became part of routine care:

*‘My team….developed systems to very rapidly get some iPads..[to] do..remote assessments….We got organised within weeks, very quickly, to be able to deliver that.’* [Consultant Psychiatrist, male, UK 4]

Several participants described how staff had to be more adaptable to meet the needs of the workplace during the pandemic. For example, a social worker described changes to their role due to ongoing staffing pressures:

*‘As nursing assistants after hours or instead of our normal work shifts, just due to the ongoing difficulties with staffing… our role has changed so markedly…. It's been trying, it's been difficult, but it's been an experience, I can tell you.’* [Social worker, male, UK 1]

Several participants noted that they felt mental health was often forgotten about in the early stages of the pandemic:

*‘In the early days, we did have to really fight the corner for mental health within … the different services and settings that we have. That..continued I guess throughout the whole vaccine rollout as well.*’ [Pharmacist, female, UK 3]

The majority of participants highlighted particular challenges they experienced in caring for patients with mental health problems during a physical health pandemic. For example:

*’COVID-19 can be associated with neutropenia, which also is something that we also look out for. So, we need to be able to juxtapose whether the neutropenia is from the COVID-19 infection or from the clozapine therapy’.* [Mental health care assistant, male, UK 1]

Several participants described how they felt that the pandemic impacted on inpatient care, mainly by slowing progress through the usual routes from acute to rehabilitation to discharge:

*‘I have seen a lot of people staying stagnant with us through the COVID period and without the opportunity to go through the rehabilitation phases. …. I think it had a big impact because people felt like they were stuck there for a very long time.’* [Consultant Psychiatrist, male, UK 1]

**THEME 2: THE NEED FOR FORMAL GUIDANCE**

Many participants commented on how they felt that their own clinical uncertainty had affected patients:

*‘The anxiety and the global situation versus the relative containment in New Zealand … created quite a dichotomy for clients who were seeing their [hospital] leave quite heavily restricted and, at the same time, not really seeing a health crisis play out locally in New Zealand’.* [Consultant Psychiatrist, male, NZ]

Several participants commented that this uncertainty could lead to patients seeking information from unreliable sources, which could serve to fuel more uncertainty and apprehension:

*‘It was interesting there [were] a number of places they [patients and the public] were getting misinformation from. Everyone was paranoid about what the viral infections could do and all sorts of interesting stories emerged’.* [Consultant Psychiatrist, male, AUS]

Some participants also described how patients often reported feeling more uncertain because of their mental health issues:

*‘Dealing with many patients who are sceptical of many things such as worldwide pandemics was very challenging. Initially many did not believe it and when we started wearing masks many became more paranoid and their mental health deteriorated’*. [Nurse, male, UK 1]

Most participants commented that they experienced the pandemic as a time of constant change:

*‘Our wards and our medical teams but also our patients needed to understand what was a really ever-changing picture.*’ [Pharmacist, female, UK 3]

Many mentioned that they felt the general guidance changed over the course of the pandemic, with frequent updates:

*‘Because it [the guidance] changed all the time, so normally if a patient got COVID, they'd send out the information because it'd be different to the last time that patient had COVID.’* [Mental health care assistant, female, UK 4]

Many reflected that in the early stages, they felt there was little formal guidance available to them and that as a result, they relied heavily on their clinical judgement:

*‘To be honest, at the very beginning, I think we went back to almost first principles. What's the pathophysiology, what's the biochemistry, and almost work forward from there, based on what we understood about disease states……At the very, very early stages, that's what it likely came down to, was independent clinical decision making’.* [Pharmacist, male, UK 2]

Many members shared their doubts about how decisions were taken and their validity:

*‘In terms of exactly what decisions were taken and how they were taken, ….A lot of staff experienced some significant doubts, on how those decisions were taken….One example was one-way systems in psychiatric wards. I mean, you're just thinking, like, 'Have you worked on a psychiatric ward?' you know, to be able to recommend this?*’. [Consultant Psychiatrist, male, UK 4]

Many participants raised concerns about the number of clinical questions which arose during the pandemic, and expressed a general feeling of uncertainty in making clinical decisions without clear guidance. Participants identified a number of priorities during the pandemic including: telepsychiatry, providing teaching for students, managing acutely unwell patients on inpatient mental health units, mental health medications, end of life care and vaccines:

*‘There were definitely areas that we were grappling with, you know, depots, clozapine, benzos, rapid tranq (sic), and also that whole, sort of, vaccine hesitancy and the confusion that came really with different groupings of who went when’.* [Pharmacist, female, UK 3]

Several participants described how decision-making felt complex in mental health settings during the pandemic, needing to balance physical and mental health causes:

*‘A lot of the patients we see do have complicated problems with thyroid, renal function complications, combination strategies, etc., and lithium's always a difficult molecule to manage in that environment.*’ [Consultant Psychiatrist, male, AUS]

Several participants described how decisions were usually made within multidisciplinary teams, but this was disrupted by COVID-19:

‘*So the way we used to …..run is like through a multidisciplinary approach but through [the] major part of the COVID we were not able to do it because people were not able to come through or were not able to get the placements’.* [Consultant Psychiatrist, male, AUS]

Several participants commented that they found formal guidance helpful in decision-making and used sources such as local pharmacy services, guidance produced by the Maudsley pharmacy services, and national guidance. In New Zealand, participants commented that patients and the public relied on updates from the government:

*‘So a lot of people, ….. use the 1:00 news updates, we made a feature of that within the unit to listen to Jacinda [Ardern, New Zealand Prime Minister] talk about what's happening around for us…..Video clips/Jacinda's news updates really helped ground and make it real for everybody.’* [Occupational therapist, female, NZ]

Several participants commented on the need to adapt general guidance for use in a particular setting or local area.

‘*But it's going to be very individualised, I think, to specific areas and what's happening. Obviously weighing up the physical environments of our wards, because they're all very different as well, …..We've got some wards, their set-out and layout is completely different, and we've noticed that in terms of some of the outbreaks that we've had on our wards’.* [Nurse matron, female, UK 2]

One consultant psychiatrist commented that guidance needed also to be broad enough to apply to all settings:

*‘The other thing is I guess your guidance has got to be applicable to every [NHS] Trust, in a way.’* [Consultant Psychiatrist, male, UK 4]

The majority of junior staff commented that they felt that decisions were and should be taken by clinicians who were more senior.

*‘Yes, I'd say, for me, as a healthcare assistant, where I was, sort of, getting my information or guidance is those higher up in management levels, rather than going out and finding it myself, it was just being given to us by the management staff.’* [Mental health care assistant, female, UK 4]

A number of participants commented that in clinical decision-making, they felt that responsibility rested with the clinician and decisions needed to be individualised. One pharmacist noted:

*‘A lot of this evidence is around supporting clinicians to make decisions, not about making them for them, so that if you're going to take a risk, you're supporting that risk.’* [Pharmacist, male, UK 2]

**THEME 3: FEEDBACK ON THE CURRENT OXPPL GUIDANCE**

Most participants had not been aware of the OxPPL guidance before the focus group. The small number who were aware had found it by chance (e.g. googling for resources on clozapine, or shared by a colleague), and several participants commented that the guidance was difficult to find:

*‘I think that just the dissemination and reach is where it could be improved. I just think it's only useful if people are actually reading it and using it.’* [Mental healthcare assistant, female, UK 4].

Some participants felt that the platform used to host the OxPPL resource was not one they were familiar with.

*‘It's not very intuitive for everything, to know what would be on there. We're not very used to the website, you know, the PPL website, so we don’t immediately think, 'Oh, let's go there.'* [Consultant Psychiatrist, male, UK 4]

Several participants commented now that they were aware, they wished they had seen the guidance earlier:

*‘So, I think it's really fantastic, and I wish, like, we'd probably had sight of some of this before to help with some issues that we'll have had in all sorts of our cases, that we've had to manage during the whole of the pandemic.’* [Nurse matron, female, UK 2]

Many participants commented that they liked the overall layout of the OxPPL resources, particularly around clinical questions, with further detail if needed:

*‘I like the way that it's laid out by clinical questions and it feels that it's had a lot of clinical input, that you've thought carefully about what is going to matter to patients, families and clinicians. So, the questions are really good. It's easy to navigate the way through it. I like the way it's not cluttered by references. So it's factual and I like the bullet points.’* [Consultant Psychiatrist, female, NZ]

Several commented that the layout and online open access made it easy to share with other staff:

*‘The layout, again, is good for dissemination among staff.….. in terms of sharing it with people as a resource, our nurses, our pharmacists, medics.’* [Pharmacist, male, UK 2]

One social worker had already shared it with colleagues and commented on its relevance:

*‘Many colleagues that I’ve forwarded it to recently have found it immensely relevant and better than NHS guidance they've been inundated with’.* [Social worker, male, UK 1]

The majority of participants commented that they felt that the methods used for the OxPPL guidance were useful and reliable:

‘*So I would see it as ….. evidence-based, it's from the University Group, this is authoritative.*’ [Consultant Psychiatrist, male, UK 4]

A number also commented on patients’ positive reactions to the methods used, which they felt provided reassurance:

‘*Patients…did appreciate the fact that there was an independent source and…. that the evidence was supporting what we were saying*’. [Consultant Psychiatrist, male, AUS]

Most participants commented they had found the OxPPL guidance to be useful and relevant:

‘*The clinical relevance is the bit that I loved the most. I had questions and I looked and there they were*.’ [Consultant Psychiatrist, female, NZ]

One nurse commented that she liked the separate sections for different professions. She said:

*‘I did notice there were sections that were relevant to specific professions, like there was nursing… I always appreciate that, to find things that are specific for what we're doing.’* [Nurse, female, NZ]

Most participants felt that the overall range of topics covered by the OxPPL guidance was good:

‘*The first thing that really struck me was the immense number of topics you've covered and the thoroughness with which they've been covered. I was really quite impressed by that. Also, some of the evidence and data that you've drawn on to substantiate various claims, again, it was very nice to see that.*’ [Consultant Psychiatrist, male, AUS]

Participants identified particular topics which they had found useful including telepsychiatry, remote cognitive testing in the elderly, inpatient and end of life care, suicide/self-harm, vaccines and vaccine hesitancy and domestic violence and abuse. For example, a nurse commented on the telepsychiatry resources:

‘*Yes, it's a very interesting area because it was an area that the nurses and the office were concerned that when we were using telepsychiatry, that it wasn't as effective and that we weren't getting the engagement.*’ [Nurse, female, NZ]

Most participants commented on the usefulness and relevance of the OxPPL guidance specifically on psychotropic medications:

‘*I remember when I wanted to join the clozapine clinic…. I got loads of documents to go through, it was so much. I felt the information we have on the [OxPPL] guidelines, is compact, something that one can go through within a few minutes…before you go in for the clinic. So I think it's much relevant to this present time that we are in*.’ [Mental healthcare assistant, male, UK 2]

Several participants also found the psychotropic medication guidance useful in their interactions with patients:

*‘So, it's useful in times of great uncertainty where people don't know what the right thing to do is and they're worried about what the risks might be to themselves, for example, about clozapine or lithium treatment*.’ [Consultant Psychiatrist, female, NZ]

Participants also commented that they felt the OxPPL guidance continued to be useful currently, even though the acute pandemic had passed. One psychiatry trainee said:

*‘So we're not in [a] pandemic now, but they're still useful.’* [Psychiatry trainee, female, UK 2].

**THEME 4: IDEAS ON FUTURE USE OF THE GUIDANCE, AND RELEVANCE TO FUTURE PANDEMICS**

Two participants suggested having a ‘champion’ in mental health teams, to increase visibility:

*‘Then maybe having a champion in each team…..[to] know the website, who knows what's on it, otherwise you're thinking, 'Oh, shall we look at that website or another one?'* [Consultant Psychiatrist, male, UK 4]

Many participants suggested adding elements to the front page for ease of use (for example, an additional explanatory video, improving the table of contents), adding more illustrations and diagrams, and changing UK specific terms to terms more understandable for the international reader:

*‘One thing I would say is the video that you had around there, which was to explain the purpose was very engaging and useful and I would actually add something in there about the scope and purpose and maybe have a 45-second clip there saying, 'This is how it's meant to be utilised. This is what you should utilise - go to first, etc.'* [Consultant Psychiatrist, male, AUS]

Most participants suggested for future use, adding new topics to those already included. Individual participants each suggested new topics (for example long COVID, teaching students in a hybrid way, creative use of staffing to meet urgent mental health needs in a pandemic, masks and engagement in mental health etc). Requests varied, many depending on the background or work setting of the participant:

*‘I guess this'll be dependent on all of our backgrounds, won't it? So yes, the bits that I found helpful were the depot, the clozapine, the lithium, the ones that we were looking at regularly at the time.’* [Pharmacist, female, UK 3]

Several participants commented that they already used the resources for patients in their clinical practice:

‘*It's useful if not to give it as a takeaway resource to say, 'Look, here's this up-to-date evidence-based guidance that says X. It's not just me making that up.' So I used it in that respect. I don't think I handed it out to anyone, but I did reference it and found it useful*.’ [Consultant Psychiatrist, female, NZ]

Several participants felt that the guidance would need significant changes to be suitable for patient use. These included reducing the length of the PDF versions, simplifying the language, and perhaps adding patient videos:

*‘It reinforces what you've said and they do remember it better because when they're reading it later on and it says the same stuff you've just read out with them, it makes a lot more impact, or they share it with their partner or someone else. I do think the language needs to be more straightforward for that and something succinct because, in our experience, if it's getting over two sides of A4, it's never going to get read, but if it's one side of A4, it's ideal.’* [Consultant Psychiatrist, male, AUS]

Several felt that COVID-19 remains a continuing priority and challenge for health services:

*‘I think personally, over the next two to three years…..COVID (will) probably remain a topical issue and a pressure in the winter*.’ [Pharmacist, male, UK 2]

The majority of participants felt that the OxPPL guidance would be helpful and necessary in the future and could be deployed quickly if needed:

*As a model, I think it's highly relevant to future pandemics or health crises and you could follow that model quite tightly and come out with something excellent much faster than you could the first time.* [Consultant Psychiatrist, female, NZ]

Others felt that more adaptations would be needed and that in general it would be easy to adapt for future pandemics:

*‘‘So, I think there is a significant role for this kind of guidance going forward…. I think if we are susceptible to one pandemic, maybe we might be susceptible to others, as well…This can be very easily translated to any respiratory airborne-contact-related pandemic, ….Hopefully, we don't, but if we need to, we can easily add the bits that are specific to that guidance and disseminate it faster, I think’.* [Consultant Psychiatrist, male, UK 1]

Several participants commented on the resources they felt were needed to prepare the OxPPL guidelines:

‘*So I think that what you'd need to do is have a critical mass of people who could quickly mobilise to generate those questions and check the specific evidence relating to those questions quickly, because there is quite a bit of intensive resourcing that needs to go in, particularly at the beginning, and then keeping something up to date to make it relevant to the front-of-mind questions that people have.*’ [Consultant Psychiatrist, female, NZ]
