# Supplementary material for: Assessing the Impact of Evidence-Based Mental Health Guidance During the COVID-19 Pandemic: Systematic Review and Qualitative Evaluation
Source: JMIR Ment Health. 2023 Dec 22;10:e52901. doi: 10.2196/52901 (PMC10760515; doi:10.2196/52901)
Supplement: Multimedia Appendix 7 [file mental_v10i1e52901_app7.docx]

**Multimedia appendix 7. Individual guidelines on mental health and COVID-19 which were not originally included in the OxPPL guidance**

The majority (24) could be excluded because the OxPPL guidance did not cover those areas, or because they were not available on line and so our methodology could not have captured them.

Those with a * were potentially eligible, but were not included in the OxPPL guidance.

| Area of mental health | Group | Publication providing information | Weblink | Date | Country | Further information |
| --- | --- | --- | --- | --- | --- | --- |
| Teleneuropsychology * | The Inter Organizational Practice Committee (IOPC) | Bilder et al [45] 2020a  Bilder et al [46] 2020b  Palmese et al [60] 2022  Postal et al [63] 2021  Scott et al [68] 2022 | <https://iopc.online/teleneuropsychology-guidelines>  [Provisional+Recommendations-Guidance+for+Teleneuropsychology-COVID-19.pdf (squarespace.com)](https://static1.squarespace.com/static/50a3e393e4b07025e1a4f0d0/t/5ebead1e378d68147eee21b4/1589554462813/Provisional+Recommendations-Guidance+for+Teleneuropsychology-COVID-19.pdf) | 2020 | USA | A coalition of representatives from national Neuropsychology organizations with advocacy expertise in the US, including: The American Academy of Clinical Neuropsychology (AACN/ American Board of Clinical Neuropsychology), Division 40 of the American Psychological Association (APA), the National Academy of Neuropsychology (NAN), the American Board of Professional Neuropsychology (ABN), the Cultural Neuropsychology Council (CNC), the American Psychological Association Services (APAS). |
| Telepsychiatry with young people * | The British Psychological Society | Schlief et al [67] 2022 | <https://www.bps.org.uk/guideline/considerations-psychologists-working-children-and-young-people-using-online-video> | 2020 | UK | Aimed at psychologists working with children and young people using online video platforms. |
| E-mental health and telepsychotherapy * | EFPA (the European Federation of Psychologists’ Associations) project group on e-health | van Daele et al [72] 2020 | <https://www.efpa.eu/recommendations-policy-and-practice> | 2020 | Europe | Recommendations for e-mental health, including telepsychotherapy. |
| Remote cognitive and behavioural assessment testing * | Alzheimer Society of Canada Taskforce on dementia care best practices in COVID-19 | Geddes et al [54] 2020 | <https://www.mcgill.ca/neuro/article/research-patient-care/remote-assessment-brain-health-during-pandemic> | 2020 | Canada | Review of available literature, and summary of guidelines (in the supplementary appendix). |
| Patients with Behavioral Health Emergencies and Suspected or  Confirmed COVID-19 * | Joint statement by the American Association for Emergency Psychiatry, American College of Emergency  Physicians, American Psychiatric Association, Coalition on Psychiatric Emergencies, Crisis Residential  Association, and the Emergency Nurses Association | Richmond et al [64] 2021 | <https://aaep.memberclicks.net/assets/joint-statement-covid-behavioral-health.pdf> | April 2020 | USA | Expert recommendations. |
| Care of adult patients with cognitive impairment requiring hospital care * | University of Queensland, Australia | Martin-Khan et al [58] 2020 | <https://chsr.centre.uq.edu.au/interim-guidance-care-adult-patients-cognitive-impairment-requiring-hospital-care-during-covid-19-pandemic-australia> | 2020 | Australia | Consensus, endorsed by the Cognitive Impairment and COVID-19 Hospital Care Guidance Readers Group and the NHMRC’s National Institute of Dementia Research (NNIDR) Special Interest Group (SIG): Cognitive Impairment Identification and Care in Hospitals |
|  | | | | | | |
| Telepsychology and CBT | - | Peros et al [61] 2021 | - | 2021 | Author is from the USA | Provides guidance points for delivering an exposure-based cognitive behavioural therapy group for adolescents and young adults via telehealth based on the authors’ experience in their clinic. |
| Narrative exposure therapy (NET) delivered remotely (e-NET) | - | Kaltenbach et al [56] 2021 | - |  | Authors are from Canada and Germany | Guidelines for delivering e-NET in patients who have experienced trauma and/or have PTSD. |
| Exposure therapy using telepsychiatry | - | Wells et al [74] 2020 | - | 2020 | Authors are from the USA | Review of guidelines with practice recommendations from the authors. |
| Telehealth guidelines for delivering CBT-ED in Eating disorders | - | Waller et al [73] 2020 | - | 2020 | International | 70 clinical colleagues approached, 22 replied and results summarised including clinical practice recommendations. |
| Eating disorders and virtual care | Canadian consensus panel | Couturier et al [51] 2021 | - | 2020 | Canada | Scoping review to assess existing guidelines, followed by COVID-19 guidelines from the group. |
| ADHD | European ADHD guidelines group (EAGG) | Cortese et al [49] 2020a  Cortese et al [50] 2020b  McGrath [59] 2020  Santosh et al [66] 2023 | <https://eunethydis.eu/eunethydis-initiatives/european-adhd-guideline-group/> | 2020 | Europe | Guidelines on the assessment and management of ADHD during the COVID-19 virus pandemic. |
| ADHD | Canadian ADHD Resource Alliance | McGrath [59] 2020 | <https://www.caddra.ca/wp-content/uploads/CADDRA-ADHD-and-Virtual-Care-FAQ.pdf> | 2020 | Canada | Guidelines and FAQs on the assessment and management of ADHD during the COVID-19 virus pandemic. |
| Psychological therapy and psychoeducation in bipolar disorder | Bipolar Disorder Program (PROMAN), University of São Paulo Medical School, São Paulo, Brazil | de Siqueira Rotenberg et al [52] 2020 | - | 2020 | Brazil | Recommendations for bipolar disorder treatment during COVID-19. |
| OCD | Working group from International College of Obsessive Compulsive Spectrum  Disorders (ICOCS) and the Obsessive-Compulsive and Related  Disorders Research Network of the European College of  Neuropsychopharmacology (OCRN) | Fineberg et al [53] 2020 | - | 2020 | International | Consensus guidelines. |
| ECT | ECT experts involved in the ‘ECT-AD’ trial | Lapid et al [57] 2020 | - | 2020 | USA | Systematic review to identify eligible papers. Consensus expert-developed guidance. |
| ECT | Royal Australian and New Zealand College of Psychiatrists (RANZCP) | Referenced in Branjerdporn et al [47] 2022 | <https://www.ranzcp.org/clinical-guidelines-publications/clinical-guidelines-publications-library/ect-treatment-during-the-covid-19-pandemic> | 2020  (Last updated August 2022) | Australia,  New Zealand | Guidelines on the provision of ECT treatment during the COVID-19 pandemic. |
| ECT | The Royal College of Psychiatrists | Referenced in Branjerdporn et al [47] 2022 | <https://www.rcpsych.ac.uk/docs/default-source/about-us/covid-19/ect-covid-19-briefing-may-20-submitted.pdf?sfvrsn=dd39d278_2> | - | UK | Recommendations in relation to continued ECT provision during the COVID-19 pandemic. |
| ECT | The Royal College of Anaesthetists (RCoA) and the Association of Anaesthetists | Referenced in Branjerdporn et al [47] 2022 | <https://icmanaesthesiacovid-19.org/anaesthesia-for-ect-during-the-covid-19-pandemic> | 2020 | UK | Guidelines for anaesthesia for ECT during the COVID-19 pandemic. |
| ECT | American Psychiatric Association | Referenced in Branjerdporn et al [47] 2022 | <https://www.psychiatry.org/file%20library/psychiatrists/apa-guidance-ect-covid-19.pdf> | 2020 | USA | Guidelines on electroconvulsive therapy as an essential procedure. |
| TMS and other types of non-invasive brain stimulation |  | Bikson et al [44] 2020 |  | 2020 | International | Survey of centres providing services and expert consensus recommendations. |
| Early intervention (EI) in psychosis | Experts from the EI service at the South London and Maudsley NHS Trust | Jauhar et al [55] 2021 | - | 2020 | UK | Consensus recommendations. |
| Intellectual disabilities |  | Alexander et al [43] 2020 | <https://radiant.nhs.uk/coronavirus-covid-19.html> |  | UK | Narrative review and expert guidelines. |
| Seclusion in youth inpatient units | Multidisciplinary group | Thompson et al [70] 2022 | - | Published 2022 | USA | Narrative review and expert guidelines. |
| Refugees and displaced people | UNHCR Regional Bureau for Middle East and North Africa | Pinzon-Espinosa et al [62] 2021 | <https://reporting.unhcr.org/sites/default/files/UNHCR%20MENA%20Mental%20Health%20and%20Psychological%20support%20during%20COVID-19%20-%20June%202020.pdf> | June 2020 | Middle East and North Africa | Report on the challenges within the relevant countries with examples of solutions and Mental health and psychosocial support (MHPSS) responses  during the COVID-19 outbreak. |
| Psychotropic drug use | - | Sabe et al [65] 2021 | - | 2020 | Not stated | Review of literature and pragmatic recommendations for the management of psychopharmacologic drugs including lithium, valproic acid, and clozapine |
| Italian mental health services | Italian Society of Epidemiological Psychiatry (SIEP) | Starace et al [69] 2020 | <https://siep.it/istruzioni-operative-siep/>  (Italian language website) | 2020 | Italy | Paper contains English summary of the guidelines, but the website is in Italian.  Guidelines aimed to reduce the spread of the COVID-19 disease within the outpatient and inpatient services affiliated with Mental Health Departments in Italy. |
| Consultation-liaison psychiatry | Academy of Consultation-Liaison Psychiatry | Tiamson-Kassab [71] 2021 | <https://www.clpsychiatry.org/news/academys-covid-19-task-force-report-published/> | 2020 | International | Summary of lessons learned and recommendations for adaptation of practice. |
| Psychiatric Staff Shortages in Emergency Settings | American Association of Emergency Psychiatry (AAEP) | Richmond et al [64] 2021 | - | 2021 | USA | Review and expert recommendations. |
| School based autism evaluations |  | Brunson McClain et al [48] 2021 |  |  | USA | Expert recommendations. |
